# Machine learning and individual variability in electrical field characteristics predict tDCS treatment response for anxiety in older adults in the ACT trial

**DOI:** 10.3389/fnhum.2026.1847131

**Published:** 2026-07-31

**Authors:** Junfu Cheng, Skylar E. Stolte, Zeyun Zhao, Andrew O'Shea, Aprinda Indahlastari, Adam J. Woods, Ruogu Fang

**Affiliations:** 1 Department of Electrical and Computer Engineering, Herbert Wertheim College of Engineering, University of Florida, Gainesville, FL, United States; 2 Center for Cognitive Aging and Memory, McKnight Brain Institute, University of Florida, Gainesville, FL, United States; 3 Department of Clinical and Health Psychology, College of Public Health and Health Professions, University of Florida, Gainesville, FL, United States; 4 J. Crayton Pruitt Family Department of Biomedical Engineering, Herbert Wertheim College of Engineering, University of Florida, Gainesville, FL, United States; 5 School of Behavioral and Brain Sciences, The University of Texas at Dallas, Richardson, TX, United States

**Keywords:** electric field modeling, finite element modeling, late-life anxiety, machine learning, transcranial direct current stimulation (tDCS)

## Abstract

**Introduction:**

Anxiety is highly prevalent in older adults and often co-occurs with neurodegenerative disorders, worsening cognitive decline and quality of life. Transcranial direct current stimulation (tDCS) has shown therapeutic potential, but outcomes remain inconsistent due to individual neurophysiological variability.

**Method:**

We trained machine learning (ML) models to predict state anxiety reduction following active tDCS paired with BrainHQ cognitive training (tDCS + CT) in older adults with moderate/severe baseline state anxiety symptoms from the Augmenting Cognitive Training in Older Adults (the ACT trial, NCT02851511). Our models predicted outcomes based on MRI-derived current density maps (J-maps) generated via finite element modeling. We evaluated the model using (1) repeated mixed-site nested cross-validation and (2) repeated cross-site held-out-site validation; in both settings, analyses were conducted on the 20 eligible participants.

**Results:**

The model achieved a mean balanced accuracy of 82% in the repeated mixed-site nested cross-validation and 72% in the repeated cross-site held-out validation. SHAP-based interpretation of our ML models identified the current density in posterior fusiform cortex and inferior temporal gyrus (posterior and temporooccipital divisions) as the most influential predictors of intervention response.

**Discussion:**

Posterior ventral temporal cortices emerged as critical substrates for predicting intervention response, consistent with prior studies linking fusiform and amygdala–temporal dynamics to anxiety severity and fearful face processing. These findings highlight how perceptual processes, particularly those mediated by the fusiform gyrus, shapes intervention response. Altogether, this work paved the way for ML-guided precision modeling to personalize non-invasive transcranial electrical interventions for anxiety in older adults.

## Graphical Abstract

**Figure:**
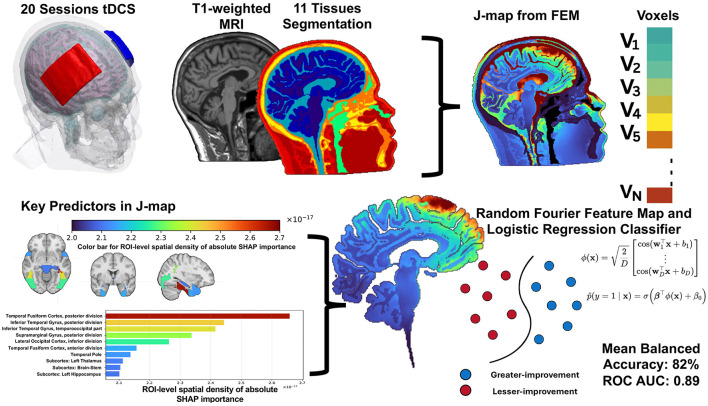

## 1 Introduction

Anxiety is a pervasive and debilitating mental health condition among aging populations (Jalali et al., 2024), frequently co-occurring with neurodegenerative conditions such as Alzheimer's Disease (Khaing et al., 2024). This co-occurrence exacerbates cognitive and emotional challenges, accelerates cognitive decline, and significantly diminishes quality of life. The cascading effects of untreated anxiety extend beyond the individual, placing increased reliance on caregivers and intensifying healthcare system burdens (Vasiliadis et al., 2013).

Emerging evidence suggests that combining transcranial direct current stimulation (tDCS) with BrainHQ cognitive training (CT) may offer a promising adjunctive approach for improving anxiety symptoms. Anxiety symptoms are commonly linked to dysregulation within prefrontal–limbic circuits, including prefrontal regulatory regions and limbic structures such as the amygdala, as well as temporal and visual–limbic pathways involved in affective stimulus processing and threat perception (Gold et al., 2015; Kenwood et al., 2022; Ressler, 2010). Hausman et al.'s research (Hausman et al., 2024) reported that participants receiving active tDCS exhibited significantly greater reductions in state anxiety symptoms compared to those receiving sham stimulation, with a 5.92 point decrease in the active group among individuals with moderate to severe baseline state anxiety (*n* = 59, *p* = 0.01). Importantly, most participants (*n* = 54) within this subgroup simultaneously underwent 3 months of training on eight adaptive BrainHQ CT tasks, suggesting a potential synergistic effect of combining tDCS and cognitive training on anxiety symptom reduction. These findings add to the growing evidence supporting the anxiolytic potential of tDCS, particularly when paired with behavioral interventions.

In a comprehensive systematic review and meta-analysis of 56 randomized, sham-controlled trials, Zheng et al. (2024) quantitatively evaluated the therapeutic effects of tDCS on depressive and anxiety symptoms across diverse clinical conditions. Based on their three-level meta-analytic model, for anxiety symptoms, Zheng et al. found a marginally significant pooled effect, with an SMD of −0.398 [*p* = 0.051, 95 % CI (−0.796, 0.001)], suggesting a trend toward anxiety reduction that did not reach statistical significance. Accordingly, Zheng et al. (2024) characterized these results as promising effects for anxiety relief. However, Zheng et al.'s multivariate meta-analysis included 16 studies (Zheng et al., 2024) also indicated the large variability across studies in the effects of tDCS on anxiety symptoms, as reflected by the significant between-study variance (*p* < 0.001).

Hausman et al.'s (2024) study and Zheng et al.'s (2024) meta-analysis indicated heterogeneity in the reported effects of tDCS on anxiety symptoms. Nevertheless, the overall promising impact of tDCS on anxiety supports the need for further investigation. Prior research, including *in vitro*, experimental, and theoretical studies, shows that the intensity of tDCS influences brain electric fields, modulates cortical excitability, and plays a key role in altering neuronal membrane potentials and synaptic plasticity (Bikson et al., 2015; Ranieri et al., 2012; Fritsch et al., 2010; Artola et al., 1990; Kronberg et al., 2017; Márquez-Ruiz et al., 2012; Podda et al., 2016). Studies have shown that higher applied current intensity correlates with greater motor evoked potential (MEP) amplitudes, indicating enhanced neuronal responsiveness in the motor cortex (Chhatbar et al., 2016; Nitsche and Paulus, 2000; Hoy et al., 2014; Esmaeilpour et al., 2018). Building on this, Evans et al. demonstrated that fixed-dose tDCS produces substantial variability in cortical electric field intensity across individuals, and Bhattacharjee et al. further showed that individualized, current-controlled dosing reduces this variability and enhances both behavioral and neurophysiologic outcomes (Evans et al., 2020; Bhattacharjee et al., 2025). Therefore, our central hypothesis is that different levels of applied current intensity in tDCS can result in varying behavioral outcomes and the heterogeneity of tDCS effect largely stems from the use of standardized, one-size-fits-all stimulation protocols that fail to accommodate the unique neuroanatomical and functional profiles of individual patients. Such conventional approaches cannot capture the considerable neurophysiological variability among individuals especially older adults, whose brain structures exhibit greater heterogeneity. In contrast, the present study explored an individual-level modeling framework that integrates each participant's T1-weighted magnetic resonance imaging (MRI) data to account for individual variations in brain morphology and electrical properties.

To understand how electrical stimulation interacts with individual brain anatomy, finite element method (FEM)-based computational modeling calculates the current density spatial distribution (J-map) of tDCS. J-map incorporates tissue conductivity through Ohm's law and therefore reflects the modeled flow of stimulation current through heterogeneous head tissues. Machine learning (ML) methods constitute an approach to investigate complex datasets. Few studies have attempted to systematically investigate estimates of tDCS J-map in human brain as predictors of responses to tDCS. This study extends conventional e-field modeling by using individualized FEM-derived stimulation-dose maps as predictive features in an ML framework. Our previous framework used ML models to predict tDCS intervention outcomes in major depressive disorder and improvements in working memory performance. This framework used tDCS J-maps derived from six-tissue segmented T1-weighted MRI data (Albizu et al., 2020, 2024). Building on this framework, the present study has three aims: (1) we aim to more accurately characterize tDCS current density distribution using 11-tissue segmented T1-weighted MRI FEM modeling; (2) we aim to explore a proof of concept for machine-learning–based prediction of individual-level tDCS response in older adults, using the spatial distribution of tDCS-induced current density during the combined tDCS and BrainHQ intervention; and (3) we aim to improve mechanistic understanding of how tDCS influences anxiety-related neural circuits in the aging brain. By integrating advanced ML algorithms with MRI-based computational models of tDCS J-map in the brain, this study suggests early feasibility of individual-level treatment outcome prediction models for dynamically optimizing and personalizing tDCS parameters to enhance tDCS therapeutic efficacy in anxiety mitigation.

## 2 Method

### 2.1 Participants and state anxiety

Augmenting Cognitive Training in Older Adults (the ACT trial, NCT02851511) recruited 379 community-dwelling older adults aged 65–89 with evidence of cognitive decline but without major neurological or medical conditions at enrollment, and assessed them at baseline, 12 weeks, and 12 months post-screening (see Woods et al., 2018 for detailed inclusion and exclusion criteria). Participants were then randomized into one of four intervention groups: tDCS + CT, Sham tDCS + CT, tDCS + Educational Training (ET) Control, and Sham tDCS + ET Control. Participants underwent a broad battery of cognitive, neuropsychological, and other measures. Within this battery, state anxiety was assessed using the State-Trait Anxiety Inventory (STAI) state score (ranging 20 to 80) in accordance with Spielberger's Manual (Spielberger, 1983). Individuals scoring ≥39 on the state subscale were categorized as having moderate/severe state anxiety symptoms and those scoring < 39 as having mild/minimal symptoms. We performed the Intent-To-Treat (ITT) analyses to assess the impact of tDCS on changes in state anxiety including six participants at the University of Florida who completed fewer than 20 sessions, while all other participants completed 20 sessions (Detry and Lewis, 2014; Hausman et al., 2023).

### 2.2 tDCS and cognitive training protocol in ACT trial

In the double-blind ACT trial, participants received either active or sham tDCS using a Soterix clinical trials stimulator during the first 20 min of 40-min CT sessions. Active tDCS delivered 2.0 *mA* for 20 min at F3 and F4 locations (cathode/anode) per the 10–20 EEG system through two biocarbon rubber electrodes, placed in saline-soaked sponges (5 × 7 *cm*^2^) presoaked with 2 mL of 0.9% NaCl, with an additional 4 mL applied to each side (for a total of 10 mL per sponge), while Sham stimulation replicated the physical sensations using a 30-s ramp-up and ramp-down with the identical electrode placement, but no current was delivered aside from the brief ramp periods following the parent ACT trial protocol (Woods et al., 2018); CT comprised 60 1-h sessions over 12 weeks using eight adaptive BrainHQ tasks targeting speed of processing and working memory (www.positscience.com), while the education control group watched 60 1 h sessions of National Geographic videos followed by quizzes over 12 weeks.

### 2.3 Data preparation and greater-improvement/lesser-improvement definition

Out of the 379 older adults from the sites of the University of Florida and the University of Arizona initially enrolled in the ACT trial, we included only those with complete STAI state scores recorded at the baseline and the post-intervention time points. Among the 302 participants meeting this criterion, 48 exhibited moderate to severe baseline state anxiety symptoms. The state anxiety scores from baseline to post-intervention among these 48 participants decreased by a median of 9.5 points. We determined greater-improvement status using a distribution-based threshold set at the sample median in STAI state score decrease from baseline assessment to post-intervention assessment among the 48 participants (Revicki et al., 2008). Participants whose reduction exceeded the median were classified as greater-improvement to the tDCS + CT intervention for state anxiety symptom improvement, while those with reductions at or below the median were considered lesser-improvement.

**Figure 1:**
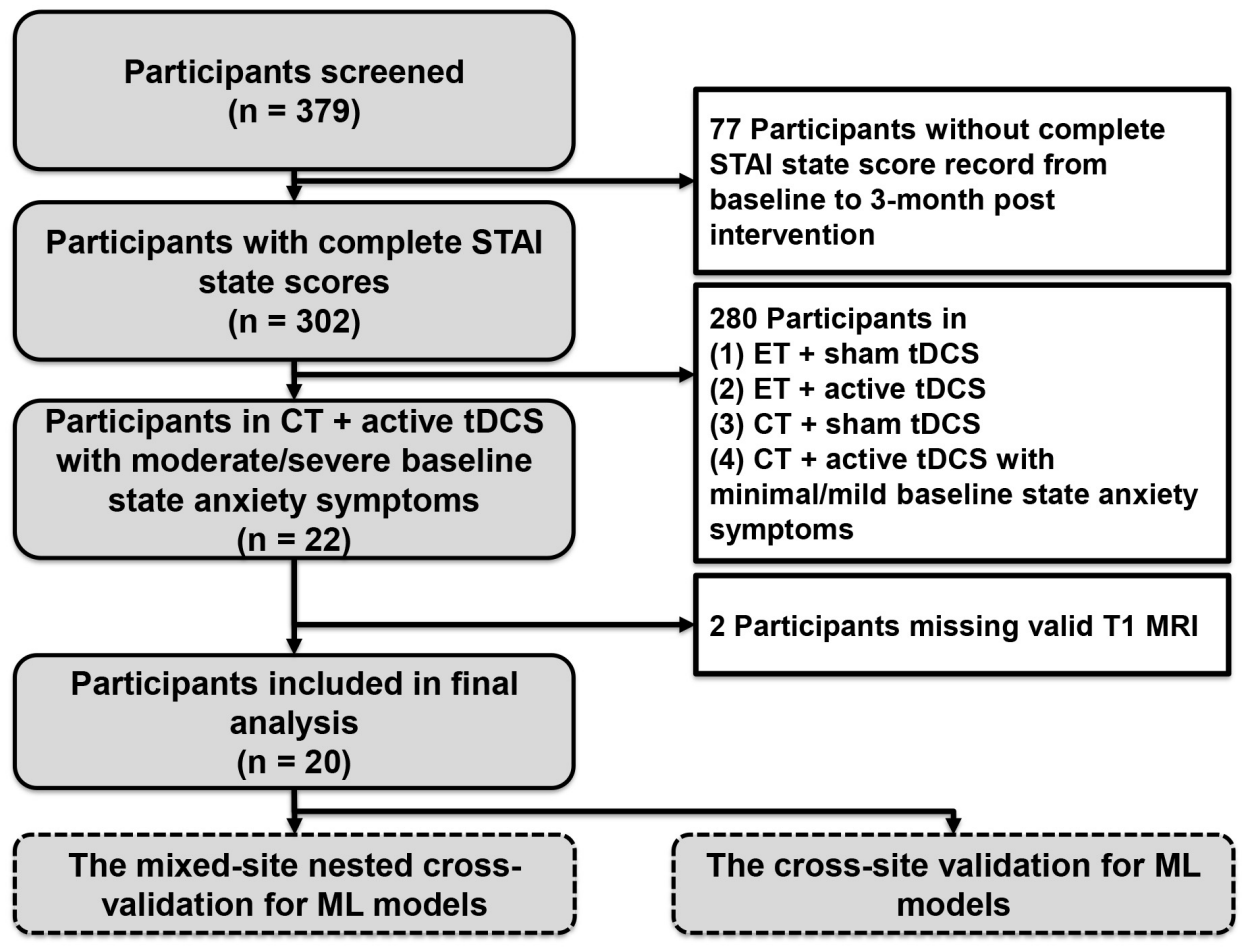
Participant flow diagram for the ACT trial analysis. We initially had a total of 379 participants on ACT trial (Hausman et al., 2024, 2023). We then excluded 77 participants due to incomplete STAI state score records from baseline to 3-month post-intervention. Out of the 302 participants with complete STAI data, we excluded 280 participants who were randomized to ET + sham tDCS, ET + active tDCS, CT with sham tDCS, or CT with active tDCS but with only minimal/mild baseline state anxiety symptoms. We identified 22 participants with moderate/severe baseline state anxiety symptoms who received active tDCS + CT, and of these, we excluded 2 participants due to missing or invalid T1 MRI data. The final dataset included 20 participants who received tDCS + CT intervention and had valid STAI state scores and MRI scans. For the present machine learning analysis, we restricted the sample to those with valid MRI data (*n* = 20) and further divided the cohort by study sites. Eleven of these 20 participants underwent intervention at the University of Florida, whereas the remaining 9 underwent intervention at the University of Arizona.

**Figure 2:**
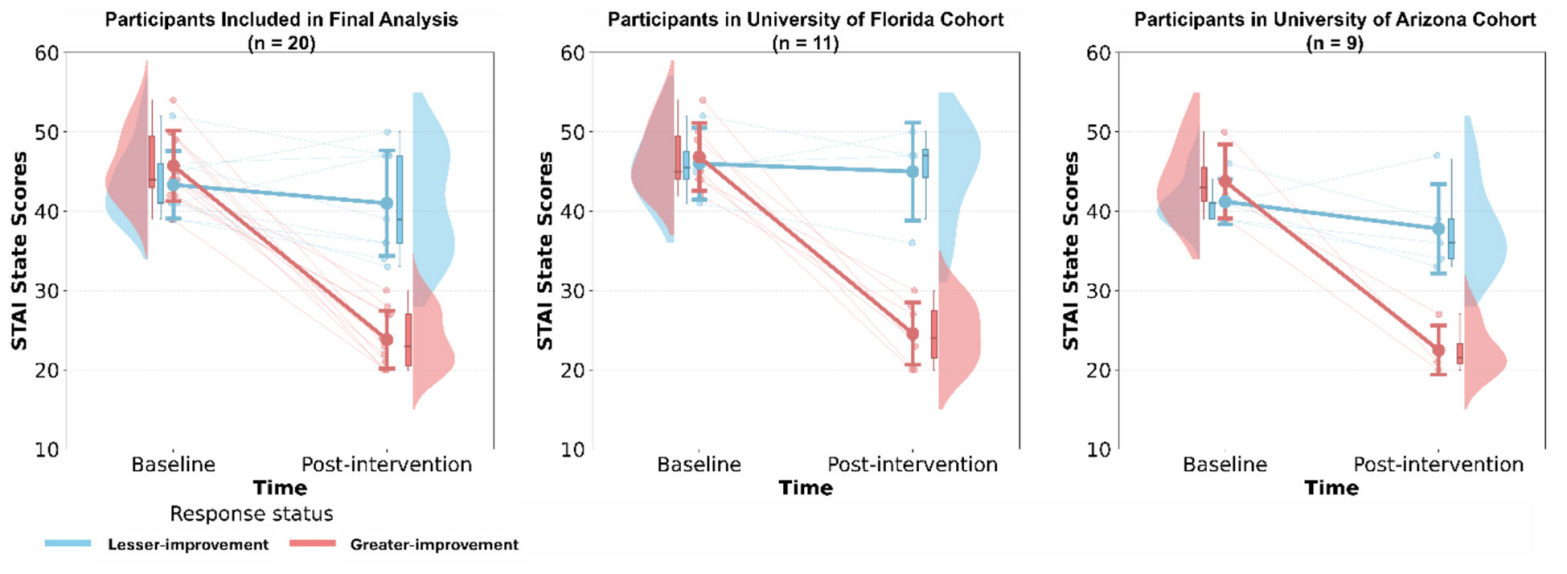
STAI state anxiety scores at baseline and post-intervention (12 weeks post-screening) by response status across full, training, and testing cohorts. Greater-improvement participants (red) demonstrated large and consistent reductions in STAI state scores following intervention, while lesser-improvement (blue) showed minimal change. Thick lines indicate group means ± standard error, thin lines represent individual trajectories, and side distributions were estimated using Gaussian kernel density estimation (KDE) with accompanying boxplots. In the University of Florida cohort, there were 4 greater-improvement participants and 7 lesser-improvement participants, while in the University of Arizona cohort, there were 5 greater-improvement participants and 4 lesser-improvement participants.

As the objective of our ML models was to predict the change in state anxiety levels among older adults with moderate/severe baseline state anxiety symptoms who received tDCS + CT intervention, we excluded participants who were not in active tDCS + CT intervention group or had minimal to mild baseline state anxiety symptoms. For the present analysis, we included only participants with valid T1-weighted MRI scans, yielding a final sample of 20. Figure 1 shows the participant selection flow. We performed greater-improvement vs. lesser-improvement comparisons of key demographic covariates, including age, sex, race, ethnicity, marital status, height, weight, BMI, and years of education, using normality-guided statistical tests; these analyses are reported in Section 1 of Supplementary Table S2 to assess whether measured baseline characteristics differed meaningfully between greater-improvement and lesser-improvement groups. STAI state score trajectories in the cohort for final analysis are illustrated in Figure 2.

### 2.4 Computational model construction and current density map generation

In the ACT trial's (Woods et al., 2018) baseline visit, structural T1-weighted magnetic resonance images (T1-weighted MRIs) were acquired using a 3-Tesla Siemens Magnetom Prisma scanner with a 64-channel head coil at the University of Florida and a 3-Tesla Siemens Magnetom Skyra scanner with a 32-channel head coil at the University of Arizona (UA). Participants were provided with earplugs to mitigate scanner noise and foam padding was used to minimize head motion. Imaging parameters were as follows: repetition time (TR) = 1800 *ms*, echo time (TE) = 2.26 *ms*, isotropic resolution = 1.0 × 1.0 × 1.0 *mm* and field of view (FOV) = 256 × 256 × 176 *mm*^3^. Of the participants who underwent T1-weighted MRI scanning (*n* = 177), 113 were scanned at University of Florida and 64 at UA. The dataset yielded an average signal-to-noise ratio (SNR) of 12.1 ± 1.54 (Stolte et al., 2024).

A team of four trained manual annotators, referred to in this paper as segmentors, segmented participants' T1-weighted MRIs into 11 tissue classes using a semi-automated labeling procedure. The 11 tissue types are white matter (WM), gray matter (GM), eyes, cerebrospinal fluid (CSF), air, major artery (Blood), cancellous bone, cortical bone, skin, fat, and muscle. The segmentation process for these 11 tissues in tDCS modeling were applied following Indahlastari et al.'s (2016) method, with modifications introduced to further enhance the quality of the results following Stolte et al.'s (2024) protocol. We manually corrected all automatic segmentation outputs in the ScanIP module in Simpleware™ software version 2018.12 (Synopsys, Inc., Mountain View, USA).

**Figure 3:**
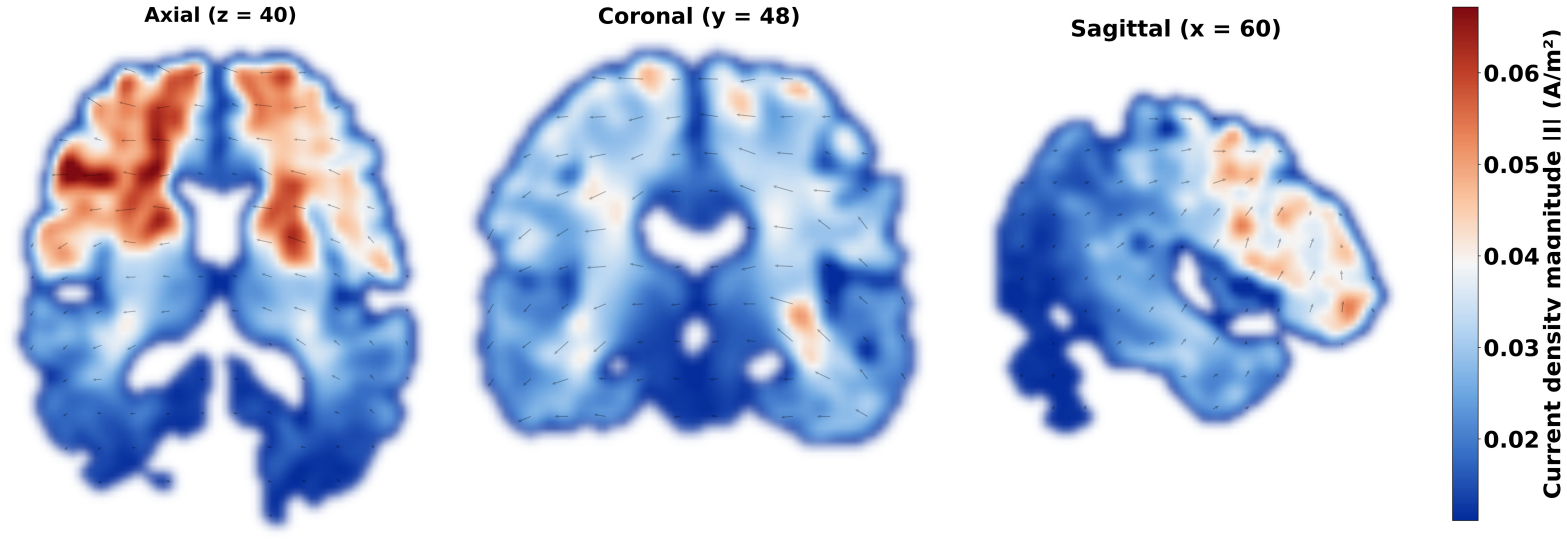
Current density distribution from an exemplary participant. The figure shows the calculated current density distribution in axial, coronal, and sagittal views for an exemplary subject. The current density maps were normalized to MNI152 space using the ICBM European-brain template in SPM12, aligning individual anatomies for subsequent feature extraction and analysis.

All tissue segmentations were then combined and visually inspected to ensure complete voxel assignment across eleven tissue classes as described above. Standard isotropic conductivity values were applied to each tissue (white matter: 0.126 *S*/*m*; gray matter: 0.276 *S*/*m*; eyes: 0.500 *S*/*m*; CSF: 1.65 *S*/*m*; air: 2.50 × 10^−14^ *S*/*m*; blood: 0.670 *S*/*m*; cancellous bone: 2.14 × 10^−2^ *S*/*m*; cortical bone: 5.52 × 10^−3^ *S*/*m*; skin: 0.465 *S*/*m*; fat: 0.250 *S*/*m*; muscle: 0.160 *S*/*m*) (Huang et al., 2019; Nielsen et al., 2018). Volumetric meshes of each tissue type were generated using iso2mesh (Fang and Boas, 2009). ROAST then applied boundary conditions, and a finite element solver (getDP Lowther and Mai, 1998) computed voltage solutions to a generalized Laplace equation within the head model. Current density *J* was derived from the simulated electric field *E* and tissue conductivity σ using Ohm's law. This voxel-wise current density, expressed in *A*/*m*^2^, quantifies the delivered stimulation dose across the whole brain. The final output of the computational model is the neuroimaging-derived J-map as shown in Figure 3. We computed all J-maps on the University of Florida's HiPerGator high-performance computing cluster, which provided access to 50 CPU cores and 175 GB of RAM.

### 2.5 Supervised machine learning models

We used a supervised machine learning framework based on J-maps derived from T1-weighted MRI scans to predict whether older adults with moderate/severe baseline state anxiety would respond to the combined tDCS and CT intervention. Specifically, we trained and evaluated separate machine learning models, each using a distinct input representation from J-maps: current density magnitude (||*J*||) alone, electric field directional encoding (θ, φ) alone, a joint directional–magnitude representation combining electric field angles and current strength (θ, φ, ||*J*||), and current density vector components aligned to anatomical axes (*J*_*x*_, *J*_*y*_, *J*_*z*_). The detailed electric field directional encoding method is described in the Supplementary material. We normalized all of these J-map–derived representations to MNI152 space using the ICBM (European brains) template in SPM12, aligning anatomies for feature extraction and analysis. To address class imbalance and enhance model performance, we ran a multi-stage preprocessing and modeling pipeline consisting of four key stages: (1) Class balancing using the Synthetic Minority Oversampling Technique (SMOTE; imbalanced-learn v0.13.0); (2) Dimensionality reduction and feature selection combining Principal Component Analysis (PCA; scikit-learn v1.6.1), Welch's t-test filtering (SciPy v1.16.0), and the Minimum Redundancy Maximum Relevance (mRMR; mrmr-selection v0.2.8) algorithm; (3) Nonlinear feature mapping via Random Fourier Features (scikit-learn v1.6.1); and (4) Classification using logistic regression trained with stochastic gradient descent (scikit-learn v1.6.1), which produced probabilistic estimates of treatment response. A detailed description of J-map direction definition and this pipeline is provided in Section 1.2 and 2.1 of the Supplementary materials. Across both mixed-site and cross-site analyses, we also trained and tested support vector regression model to predict participants' change of STAI state score. To assess whether J-map–based prediction was influenced by potential demographic and anthropometric confounders, we compared models using J-map features alone with models incorporating J-map features plus covariates including age, sex, marital status, race, height, weight, body mass index, and years of education.

### 2.6 Machine learning model training and testing

We evaluated model generalizability using two distinct machine learning validation strategies: (1) repeated mixed-site nested cross-validation approach and (2) repeated cross-site held-out validation approach, implemented separately to ensure the robustness and independence of performance estimates. In the mixed-site approach, we conducted 20 repetitions of five-fold nested stratified cross-validation among participants with moderate to severe baseline state anxiety who received the combined tDCS + CT intervention at both the University of Florida and the University of Arizona. In the cross-site approach, data from the University of Florida served as the training set, whereas data from the University of Arizona constituted the testing set, enabling an independent evaluation of the model's generalizability across distinct research sites.

We trained the models on the training set using three-fold cross-validation, and optimized hyperparameters via Bayesian optimization using a Gaussian Process surrogate model with a Matérn kernel. The entire pipeline was repeated under 20 random seeds, generating 20 models. We assessed model performance on the testing set. We developed a *post-hoc* interpretation framework that generates voxel-level SHAP attributions in MNI152 space using the Python SHAP package. We aggregated voxel-level absolute SHAP values within anatomical regions of interest (ROIs) defined by the Harvard–Oxford atlas, resulting in ROI-Level Spatial Density of absolute SHAP Importance. For each ROI, the sum of absolute SHAP values was normalized by voxel count to account for differences in region size. We present a comprehensive description of ML model training, testing, and interpretation in Section 2.2, 2.3, and 3 of the Supplementary materials.

## 3 Results

### 3.1 Machine learning model performance report

**Figure 4:**
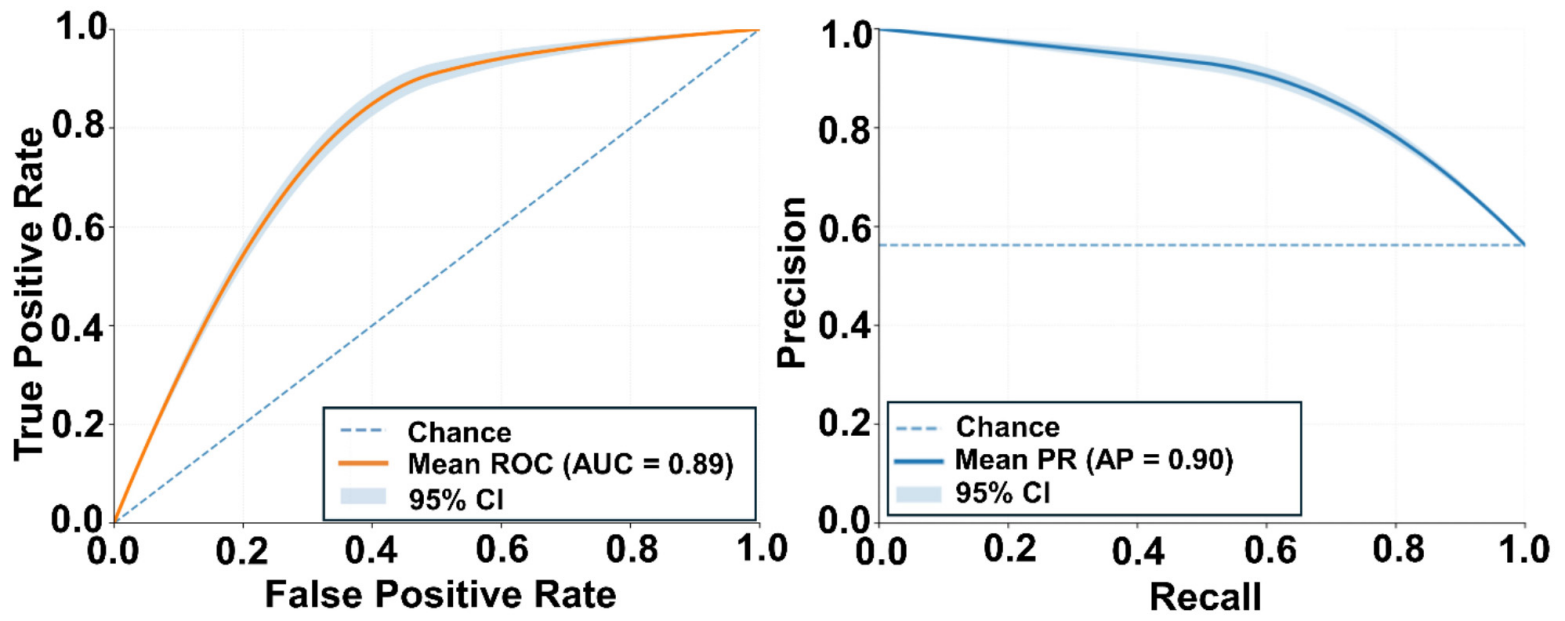
Aggregated ROC and PR curves for the repeated mixed-site nested cross-validation approach. The **(left panel)** shows the mean ROC curve (area under the curve, AUC = 0.89) with 95% CI bands. The **(right panel)** shows the mean PR curve (average precision, AP = 0.90) with 95% CI.

Among all models evaluated using different J-map–derived feature representations, the current density magnitude (||*J*||) model achieved the best performance under the repeated mixed-site nested cross-validation approach. As shown in Table 1 and Figure 4, the model with current density magnitude (||*J*||) input demonstrated the highest performance using mixed-site approach (balanced accuracy = 82%; receiver operating characteristic area under curve, AUROC = 0.89) and remained robust under the repeated cross-site held-out validation approach (balanced accuracy = 72%; AUROC = 0.74). The mean true-positive rate of 81% in the cross-site approach indicated that the model can reliably identify older adults who are likely to respond to the tDCS + CT intervention, even when tested on data from a distinct recruitment site. Detailed performance results for machine learning models trained on other J-map–derived feature representations are provided in Supplementary material Section 3.1.

**Table 1:** Model performance metrics on the testing set in the repeated mixed-site nested cross-validation approach and the repeated cross-site held-out validation approach.

Validation strategy	Metric	Mean ±SD	95% CI (Lower–upper)
Mixed-site nested cross-validation	AUPRC	0.90 ± 0.10	0.89–0.92
	AUROC	0.89 ± 0.11	0.86–0.91
	Balanced Accuracy	0.82 ± 0.16	0.79–0.85
	Weighted F1	0.81 ± 0.17	0.78–0.84
	PPV	0.88 ± 0.14	0.85–0.91
	TPR	0.82 ± 0.23	0.77–0.86
	MCC	0.67 ± 0.29	0.61–0.73
Cross-site held-out validation	AUPRC	0.73 ± 0.12	0.68–0.78
	AUROC	0.74 ± 0.12	0.69–0.78
	Balanced Accuracy	0.72 ± 0.06	0.70–0.74
	Weighted F1	0.70 ± 0.06	0.68–0.73
	PPV	0.67 ± 0.13	0.62–0.72
	TPR	0.81 ± 0.16	0.75–0.88
	MCC	0.47 ± 0.13	0.43–0.52

Positive Predictive Value (PPV); True Positive Rate (TPR); Weighted F1 Score (Weighted F1); Matthews Correlation Coefficient (MCC); area under the receiver operating characteristic curve (AUROC); area under the precision–recall curve (AUPRC). Values are reported as mean ± standard deviation (SD). The 95% confidence interval (CI) indicates the range for the mean across runs.

In addition to models evaluated using J-map magnitude feature representations, the performance of ML model using current density with other J-map–derived feature representations is demonstrated in Table 2. Models based on directional features or current vector components showed consistently lower performance, particularly under cross-site validation. The superior performance of the current-density magnitude model suggests that individual differences in the spatial distribution and strength of stimulation may be more informative for predicting anxiety improvement than directional encoding alone. The modest reduction in performance when directional features were added may reflect increased feature dimensionality relative to the small sample size, additional noise introduced by angular estimates, or overfitting due to redundant directional information.

**Table 2:** Performance of machine learning models using different J-map–derived feature representations under mixed-site nested cross-validation and cross-site held-out validation strategies.

Feature representation	Validation strategy	AUPRC	AUROC	Balanced accuracy	Weighted F1	MCC
**Current density magnitude (||J||)**	Mixed-site Nested Cross-validation	**0.90** **±0.10**	**0.89** **±0.11**	**0.82** **±0.16**	**0.81** **±0.17**	**0.67** **±0.29**
Current density direction (θ, ϕ)		0.44 ± 0.23	0.60 ± 0.13	0.50 ± 0.15	0.42 ± 0.17	0.01 ± 0.35
Directional field + magnitude (θ, ϕ, ||J||)		0.54 ± 0.26	0.68 ± 0.18	0.50 ± 0.16	0.44 ± 0.18	0.00 ± 0.35
Current vector components (Jx, Jy, Jz)		0.54 ± 0.21	0.67 ± 0.16	0.53 ± 0.19	0.48 ± 0.21	0.06 ± 0.43
Current density magnitude (||J||)	Cross-site Held-out Validation	0.73 ± 0.12	0.74 ± 0.12	0.72 ± 0.06	0.70 ± 0.06	0.47 ± 0.13
Current density direction (θ, ϕ)		0.50 ± 0.12	0.57 ± 0.08	0.49 ± 0.09	0.37 ± 0.11	0.00 ± 0.24
Directional field + magnitude (θ, ϕ, ||J||)		0.58 ± 0.13	0.62 ± 0.14	0.55 ± 0.11	0.45 ± 0.14	0.14 ± 0.30
Current vector components (Jx, Jy, Jz)		0.35 ± 0.21	0.46 ± 0.13	0.41 ± 0.13	0.31 ± 0.13	−0.22 ± 0.28

Weighted F1 Score (Weighted F1); Matthews Correlation Coefficient (MCC); area under the receiver operating characteristic curve (AUROC); area under the precision–recall curve (AUPRC). Values are reported as mean ± standard deviation (SD). Bold values indicate the best performance within each validation strategy.

### 3.2 Voxel-wise model interpretation results

**Figure 5:**
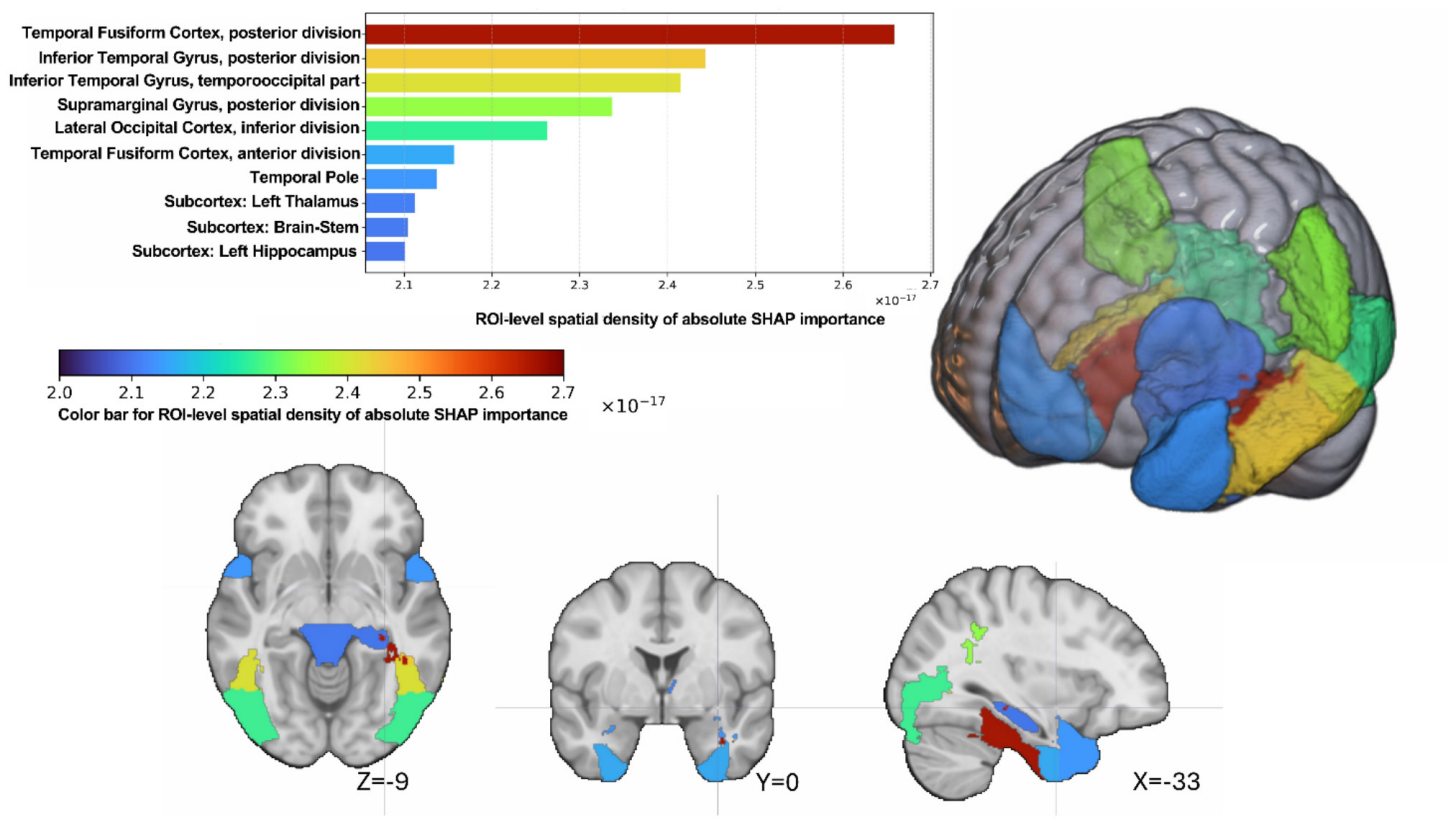
Top 10 ROI-level spatial density of absolute SHAP importance. Top 10 ROI-level spatial density of SHAP importance map in whole brain. Visualizations were generated using MRIcroGL v26.0.1 (https://www.nitrc.org/), with overlays mapped onto the MNI152 template supplied by the software. Mean absolute SHAP value across voxels per ROI displayed on the MNI152 template. This highlighted the most influential brain regions identified by the model. The absolute SHAP magnitudes (≈10^−7^) were numerically small because each voxel contributes only a minute fraction of the model's output probability after dimensionality reduction and back-projection. During PCA, principal components represented linear combinations of thousands of voxels; when SHAP values computed in this reduced space are mapped back to voxel space, their magnitudes were proportionally diluted. Given that the J-map contained nearly 600,000 voxels and most exhibit near-zero contribution. Warmer colors indicate regions of interest (ROIs) with current density distribution having stronger contributions to the model. Overall, these results suggested that ventral visual/temporal areas, especially the posterior fusiform and the inferior temporal cortex, were key in determining who benefits from combined tDCS and BrainHQ CT intervention. Other ROIs (e.g., superior frontal gyrus, precentral gyrus) exhibited lower spatial densities of SHAP importance.

**Figure 6:**
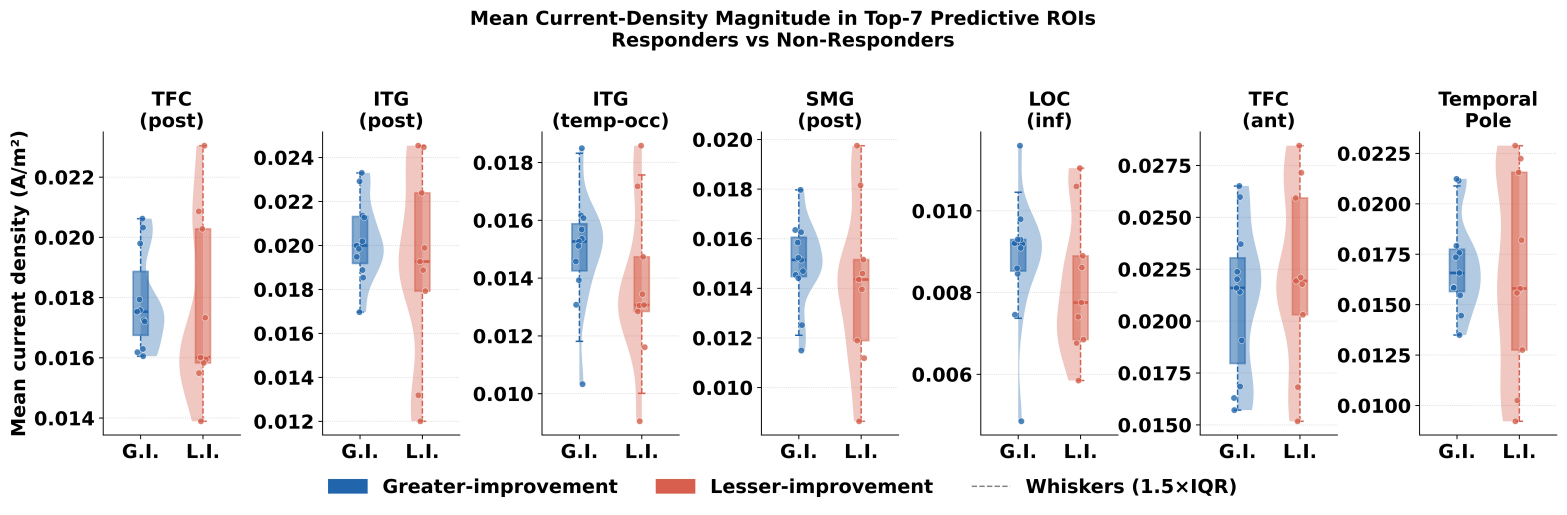
Distribution of mean current density magnitude within the top seven predictive ROIs by Response Group. Box plots show the average current density magnitude within the seven ROIs with the highest spatial density of absolute SHAP importance, stratified by greater-improvement participants and lesser-improvement participants. TFC, temporal fusiform cortex; ITG, inferior temporal gyrus; SMG, supramarginal gyrus; LOC, lateral occipital cortex; post, posterior division; temp-occ, temporooccipital part; inf, inferior division; ant, anterior division; G.I., greater-improvement; L.I., lesser-improvement. Whiskers represent 1.5× the interquartile range. These plots summarize modeled current-density magnitude, not neural activity, and are intended to descriptively illustrate regional stimulation-dose variability across response groups.

Based on the spatial density distribution of absolute SHAP values, the top seven regions with the highest spatial density of SHAP importance were all located in the cortex. The posterior division of the temporal fusiform cortex exhibited the highest concentration of predictive importance, with a markedly higher mean of absolute SHAP values than most other regions. These observations identified it as the most influential region in predicting response to tDCS + CT. Following close behind were the inferior temporal gyrus, posterior division, and inferior temporal gyrus, temporooccipital part. Overall, current density in posterior ventral temporal regions exhibited a high spatial density of predictive importance (see Figure 5 for the top 10 contributing ROIs). All 34 ROI-level spatial density of absolute SHAP importance with non-zero values and the current intensity spatial distribution from the 20 participants' J-map are represented and visualized in the Supplementary material. Importantly, these SHAP-derived values indicate the relative contribution of regional current-density features to the model's classification decisions, but they do not directly indicate increased or decreased neural activity in these ROIs. To descriptively examine whether the top predictive ROIs showed different modeled stimulation-dose patterns between response groups, we compared the distribution of average current-density magnitude between greater-improvement participants and lesser-improvement participants (Figure 6). The two groups showed substantial overlap across ROIs, although lesser-improvement participants demonstrated greater variability and higher upper-range modeled current-density values in several regions. Therefore, these distributions should be interpreted as descriptive evidence of regional current-density heterogeneity rather than as definitive directional biomarkers of response or non-response.

### 3.3 Prediction of STAI state-score decrease using J-map feature representations

Support vector regression model testing showed limited predictive performance for estimating STAI state score decrease from baseline to 12 weeks using J-map feature representations, as shown in Table 3. Across both validation strategies, all feature representations produced negative R^2^ values, indicating that the models did not outperform a mean-prediction baseline. In the cross-site analysis, performance was relatively similar across representations, with current density magnitude (||J||) yielding the lowest MAE and RMSE (9.25 ± 0.04 and 10.86 ± 0.05, respectively) and sharing the least negative R^2^ with the magnitude-combined representations. Direction-only features showed the weakest cross-site performance. In the mixed-site analysis, prediction errors were larger overall, with directional field plus magnitude (θ, ϕ, and ||J||) producing the lowest MAE and RMSE (11.24 ± 1.79 and 12.56 ± 1.84, respectively), while both this representation and current vector components (Jx, Jy, and Jz) showed the least negative R^2^ values. Overall, these findings suggest that J-map-derived current-flow features did not provide robust predictive accuracy for STAI state score reduction, although small feature-dependent differences were observed.

**Table 3:** Mixed-site and cross-site prediction of STAI score decrease using J-map feature representations with support vector regression.

Feature representation	Validation strategy	MAE, mean ±SD	RMSE, mean ±SD	R^2^, mean ±SD
**Current density magnitude**, **||J||**	Cross-site	**9.25** **±0.04**	**10.86** **±0.05**	**−0.13** **±0.01**
Current density direction, θ and ϕ		9.27 ± 0.09	10.89 ± 0.11	−0.14 ± 0.02
Directional field + magnitude, θ, ϕ, and ||J||		9.26 ± 0.06	10.87 ± 0.07	−0.13 ± 0.01
Current vector components, Jx, Jy, and Jz		9.26 ± 0.01	10.87 ± 0.09	−0.13 ± 0.02
**Current density magnitude**, **||J||**	Mixed-site	11.29 ± 1.88	12.60 ± 1.91	−0.85 ± 1.61
Current density direction, θ and ϕ		11.26 ± 1.78	12.59 ± 1.81	−0.85 ± 1.57
Directional field + magnitude, θ, ϕ, and ||J||		11.24 ± 1.79	12.56 ± 1.84	−0.83 ± 1.55
Current vector components, Jx, Jy, and Jz		11.28 ± 1.79	12.59 ± 1.83	−0.83 ± 1.52

Bold values indicate the best performance within each validation strategy.

### 3.4 Model performance after adding confounder variables

As shown in Supplementary Table S4, adding confounder variables consistently reduced model performance across both mixed-site and cross-site validation. The J-map-only models outperformed the confounder-augmented models across all evaluation metrics, indicating that the predictive signal was not improved by inclusion of measured confounders. These findings suggest that J-map features retained stronger predictive value on their own and that the observed performance was not primarily driven by simple demographic or clinical covariates. The inclusion of demographic variables led to a marked performance collapse, indicating limited robustness and a high susceptibility to noisy inputs.

## 4 Discussion

In this study, we trained and tested ML models based on J-maps to predict state anxiety response among older adults with baseline moderate to severe state anxiety who underwent active tDCS combined with BrainHQ CT. Our supervised pipeline was specifically designed for high-dimensional neuroimaging data, incorporating spatial normalization and careful feature reduction. The models demonstrated distinct performance across validation strategies; the models achieved a mean balanced accuracy of 82% in the repeated mixed-site nested cross-validation approach and 72% in the repeated cross-site held-out validation approach. The lower cross-site performance likely reflects site-related distribution shifts, differences in MRI data acquisition, and a limited sample size, while still supporting preliminary generalizability. From a clinical perspective, this level of performance suggests preliminary potential for individualized response prediction, although the present model is not yet ready for clinical decision-making and requires validation in larger, independent, multisite samples. These findings are clinically meaningful because they suggest a future workflow in which pretreatment MRI data could be used to generate individualized current-density maps, and a validated ML model could then estimate the likelihood of response to a specific tDCS + CT protocol before treatment begins. In this proposed workflow, current-density modeling alone would describe the individualized stimulation-dose distribution, whereas ML would be needed to link that distribution to the probability of clinical response. Therefore, the translational value of the present approach lies in combining individualized FEM-based dose modeling with outcome-prediction models, rather than using regional current-density estimates alone as clinical decision rules. In future studies, such a framework could be extended to compare alternative current intensities or electrode placements and identify stimulation parameters that are more likely to benefit a given individual. For example, if a validated model predicted low likelihood of response to the standard F3/F4 montage, future optimization studies could evaluate whether an alternative montage or individualized current dose better engages anxiety-relevant circuits. Moreover, by leveraging neuroimaging-derived current density maps, the model provides insight into the neurophysiological underpinnings of intervention response, advancing precision medicine approaches for anxiety in older adults.

A voxel-wise SHAP attribution method identified regional current-density features that contributed to model predictions. Current-density features in the temporal fusiform cortex, posterior division, emerged as the most influential contributors to the model, followed by features in the inferior temporal gyrus, posterior division and temporooccipital part. These findings suggest that individual variability in modeled current-density distribution within posterior ventral temporal regions contributed to response prediction, although they do not establish whether neural activity in these regions increased or decreased after intervention. Although frontal regions are commonly implicated in cognitive control and emotion regulation, they did not rank among the highest predictive contributors in the present model. This should not be interpreted as evidence that frontal regions are unimportant for anxiety response. Because all participants received the same DLPFC montage, frontal current-density features may have shown comparatively less between-subject variability, limiting their contribution to prediction. Future studies including sham stimulation, alternative montages, and larger samples are needed to determine the causal and predictive roles of frontal regions.

The prominent contribution of fusiform current-density features is notable given the fusiform gyrus's central role in perceptual processing within the ventral visual pathway, where it supports the transformation of complex sensory inputs into coherent object representations. Kanwisher and Yovel demonstrated that the fusiform face area (FFA), located within the fusiform gyrus, is selectively engaged in face perception, supporting domain-specific processing of complex visual categories (Kanwisher and Yovel, 2006). Extending this view, Bilalić found that the FFA contributes importantly to the perceptual mechanisms underlying visual expertise, suggesting that fusiform gyrus activity generalizes to higher-order visual discrimination and pattern integration beyond faces, reflecting perceptual learning processes (Bilalić, 2016). Weiner and Zilles (2016) emphasized the fusiform gyrus's anatomical–functional specialization for high-level vision including all of face, object, and word recognition. These findings imply that repeated visual discrimination training, such as that provided by the BrainHQ cognitive training modules including Visual Search, Divided Attention, Multiple Object Tracking (MOT), and Useful Field of View (UFOV) may engage and strengthen fusiform gyrus-mediated perceptual networks that support rapid visual categorization and attention allocation. This interpretation aligns with the two-visual-systems hypothesis outlined by Foley et al. (2015), which situates the fusiform gyrus within the ventral stream responsible for allocentric, perceptual representations critical for conscious perception and perceptual updating. Furthermore, Ho et al. (2016) reported that fusiform activity correlates with perceptual processing efficiency during affective face perception in adolescents with depression, linking fusiform gyrus function to adaptive perceptual precision in emotionally salient contexts. Accordingly, repeated engagement of these perceptual circuits during cognitive training may enhance fusiform-dependent visual efficiency and attentional filtering, facilitating more stable and adaptive interpretation of environmental stimuli. However, because the present study did not directly measure task-related neural activity or pre-post functional changes, this proposed perceptual mechanism remains speculative and should be tested in future multimodal studies. This theory offers a plausible neurocognitive pathway through which combined tDCS and cognitive training promote state anxiety reduction.

Li et al.'s (2019) study provided direct neuroimaging evidence that supports our interpretation: higher state anxiety in otherwise healthy participants correlates with increased inferior temporal gyrus activity, both in resting-state measures (ALFF) and in the context of prior task-based findings. Specifically, they reported positive correlations between state anxiety scores and the amplitude of low-frequency fluctuations (ALFF) values in both the left and right inferior temporal cortex [left: MNI (−42, −9, −33); *r* = 0.43, *p* < 0.001; right: MNI (60, −36, −21); *r* = 0.37, *p* < 0.001], indicating that higher self-reported state anxiety was associated with greater spontaneous neural activity in this region. Functionally, Li et al. argued that the inferior temporal gyrus is engaged in emotional working memory and face/emotion recognition, and that its heightened activity in high-anxious individuals likely reflects increased sensitivity to emotional stimuli. Taken together, these converging lines of evidence reinforce the idea that posterior ventral temporal regions, particularly the inferior temporal and fusiform gyri, represent critical substrates for both the maintenance of anxiety states and their modulation through targeted interventions.

Consistent with these SHAP-derived findings, Jung et al. (2018) reported that resting-state functional connectivity (rs-FCN) between the amygdala and posterior temporal cortices is critically involved in anxiety pathology. Specifically, they observed that abnormal positive coupling of the left amygdala with the precuneus and supramarginal gyrus was associated with higher Hamilton Anxiety Scale scores, indexing general anxiety. Moreover, connectivity between the right amygdala and middle temporal gyrus was reduced in patients with social anxiety disorder compared with controls, and this disruption correlated inversely with social anxiety symptom severity (Liebowitz Social Anxiety Scale and Social Phobia Scale). Additional alterations in inferior and ventral temporal regions, including the fusiform and superior temporal gyri, were also linked to symptom burden. These converging results suggest that aberrant amygdala–temporal and amygdala–default mode network (DMN) interactions underlie heightened emotional reactivity and symptom severity in anxiety disorders, reinforcing the importance of posterior ventral temporal cortices identified by our predictive model.

In addition, Frick et al. (2013) demonstrated that patients with social anxiety disorder (SAD) exhibit heightened posterior fusiform responses to fearful faces, with significantly greater activation relative to healthy controls in both the right fusiform gyrus [*Z* = 3.31, *p* < 0.001, Volume: 1,053 mm^3^, MNI (45, −52, −20)] and the left fusiform gyrus [*Z* = 3.30, *p* < 0.001, Volume: 513 mm^3^, MNI (−42, −67, −20)]. Moreover, psychophysiological interaction analyses revealed increased functional connectivity between the fusiform gyrus and the right amygdala (*Z* = 3.13, family-wise error-corrected *p* = 0.04) during fearful face processing, indicating an abnormal coupling between regions supporting visual processing and fear reactivity. Importantly, within the SAD group, anxiety severity (LSAS-SR score) positively correlated with amygdala–right fusiform gyrus connectivity (*Z* = 3.79, *p* < 0.001), further linking posterior fusiform involvement to clinical symptomatology. Together, these results reinforce our model-derived finding that posterior fusiform regions serve as critical predictors of anxiety outcomes, suggesting that altered fusiform–amygdala dynamics may provide a mechanistic substrate through which perceptual and affective biases are targeted by intervention combined tDCS and CT.

**Figure 7:**
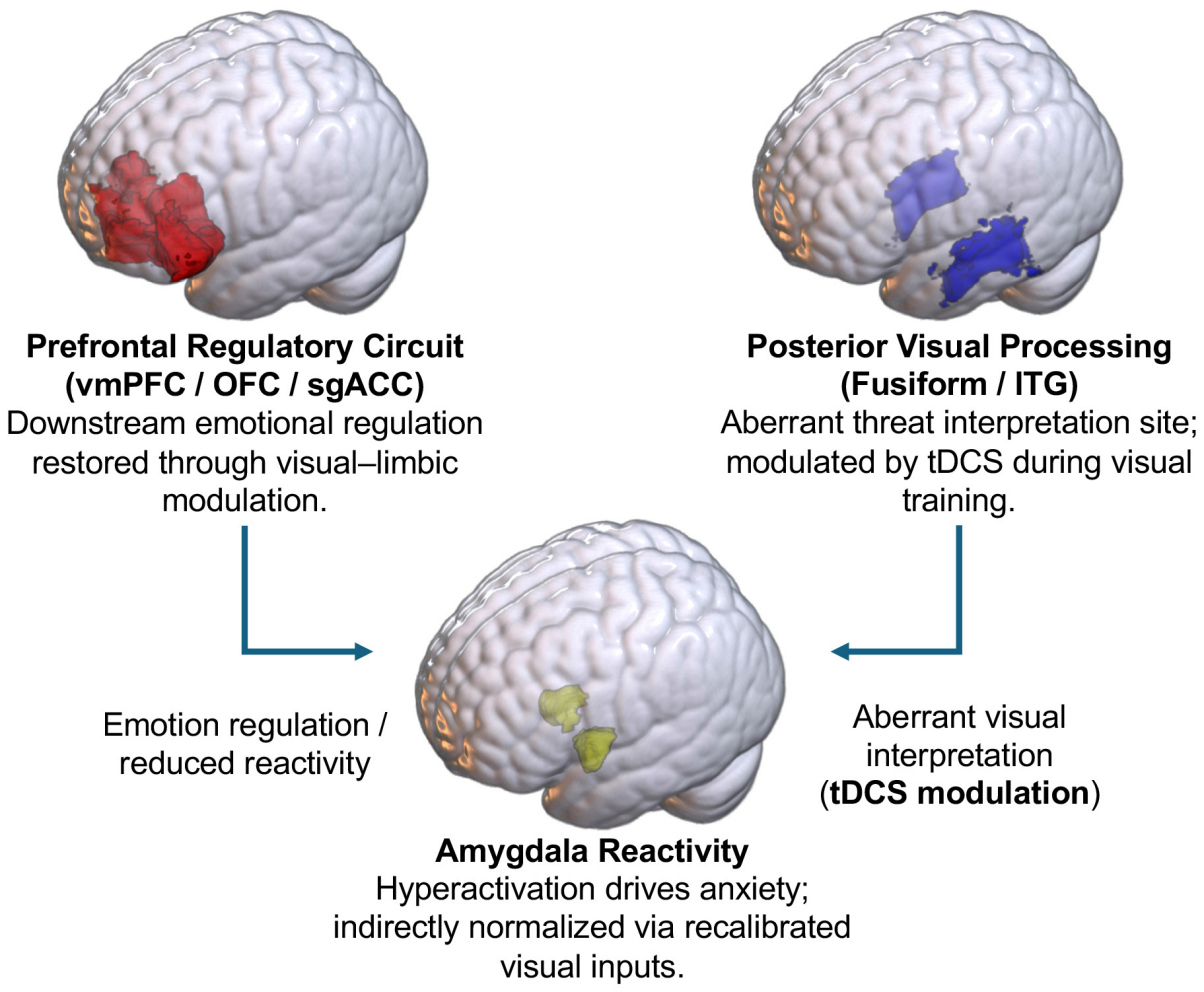
Proposed mechanism of tDCS modulation of state anxiety symptoms. tDCS may reduce anxiety by recalibrating visual–limbic coupling rather than relying solely on prefrontal regulatory pathways involving the ventromedial prefrontal cortex (vmPFC), orbitofrontal cortex (OFC), and subgenual anterior cingulate cortex (sgACC). Modulation of the posterior fusiform cortex and inferior temporal gyrus (ITG) helps correct threat misinterpretation, normalizing amygdala reactivity.

Building upon this observation, we propose a hypothesis-generating model in which individual variability in tDCS-induced current distribution may relate to anxiety symptom improvement through visual–limbic pathways, rather than through prefrontal control mechanisms alone (Figure 7). The posterior fusiform cortex and inferior temporal gyrus serve as visual processing hubs. Aberrant interpretation of these cues may elicit excessive amygdala activation, contributing to heightened anxiety responses. Through concurrent engagement of posterior perceptual regions during BrainHQ CT, individual differences in tDCS current distribution may influence visual–limbic processing of threat-related information; however, the present data cannot determine whether amygdala activity or regional neural activation increased or decreased after intervention.

Although electrodes were placed over frontal regions, modeled tDCS current density was not limited to the cortex directly beneath the electrodes. In individualized FEM models, current flow depends on both electrode placement and subject-specific head conductivity. Therefore, posterior temporal and fusiform regions may still receive measurable current flow and be modulated. Their involvement may reflect effects on visual emotional processing during the combined tDCS and cognitive training intervention. Current-density distributions for these regions are shown in Supplementary materials.

The intended translational use of this approach is not current-density modeling alone, but a combined FEM + ML precision-treatment workflow. In this future workflow, pretreatment MRI data would be used to generate individualized current-density maps for a candidate tDCS montage, and a validated ML model would estimate the likelihood of clinical response to that specific protocol. If validated prospectively, this framework could support treatment planning by identifying individuals likely to respond to the standard montage and by motivating alternative montage or dose-optimization strategies for individuals predicted to have a low likelihood of response. However, the present proof-of-concept model is not yet ready for clinical decision-making, and larger sham-controlled and multisite studies are needed to establish calibration, decision thresholds, and clinical utility.

Despite encouraging predictive performance, several limitations prevent direct clinical use of the present model. The small sample size limits generalizability, increases overfitting risk, and prevents establishing clinically actionable prediction thresholds. Future work should evaluate model calibration, decision thresholds, and prospective clinical utility before this approach is used to guide individual treatment selection. Because of the small sample size, particularly in the cross-site held-out testing cohort, we did not perform formal greater-improvement vs. lesser-improvement inferential tests on ROI-level SHAP values. The testing set included only 9 participants, with 5 greater-improvement participants and 4 lesser-improvement participants, making non-parametric group comparisons highly underpowered and sensitive to individual observations. Therefore, SHAP-based ROI findings were interpreted as *post-hoc* model explanations rather than independent group-level neurobiological measurements. These findings should be considered exploratory and hypothesis-generating, and future larger studies are needed to determine whether ROI-level SHAP patterns or regional current-density features differ reliably between greater-improvement participants and lesser-improvement participants. The reported model performance should be interpreted as preliminary and that SHAP-derived spatial patterns may be unstable in this sample. Cross-site held-out validation were included to evaluate whether model performance was driven by site-specific patterns. However, because each site contributed a limited number of participants, these analyses should be interpreted as preliminary. Isotropic conductivity assumptions in this study may introduce modeling error in older adults, and future studies should incorporate diffusion MRI-informed anisotropic conductivity modeling when available. Additionally, deep learning approaches were not explored; future work could apply convolutional or graph-based models to capture complex neuroimaging patterns and improve accuracy. Longitudinal and multimodal studies integrating genetics, behavior, and physiology may further advance precision treatment strategies for anxiety in older adults.

Because the present ML analysis was restricted to participants receiving active tDCS + CT, the identified J-map predictors cannot yet be used to determine whether an individual should receive active tDCS + CT rather than sham, cognitive training alone, or another intervention. The observed predictive features may partly reflect general prognostic neuroanatomical or clinical characteristics associated with anxiety improvement during cognitive training. Future work should directly compare active and sham tDCS conditions using parallel modeling pipelines to determine whether J-map-derived predictors explain variance uniquely attributable to active stimulation and whether they improve treatment-selection decisions beyond clinical and demographic predictors alone.

In summary, our findings show that ML models using neuroimaging-derived current-density maps show preliminary ability to predict state anxiety treatment response to tDCS combined with BrainHQ CT in older adults. Posterior ventral temporal regions, especially the fusiform and inferior temporal gyri, emerged as key current-density-based contributors to model prediction, highlighting their potential relevance for understanding individual variability in anxiety response to tDCS + CT. These data provide a foundation for future clinical trials attempting to selectively neuromodulate activity within the identified brain regions to improve anxiety symptoms. In addition, this work provides foundational evidence for future studies testing a precision-treatment workflow that combines individualized finite-element current-density modeling with validated ML outcome prediction. In such a workflow, FEM modeling would quantify individualized stimulation-dose distributions, while ML prediction would estimate the likelihood of clinical response and support future montage or dose optimization. However, given the small sample size these findings require validation in larger and independent samples from moderate/severe state anxiety older adult cohort before conclusions can be drawn regarding treatment-specific prediction, clinical utility, or personalized stimulation targeting.

## Data Availability

The data analyzed in this study is subject to the following licenses/restrictions: Data are managed under the data sharing agreement established with NIA and the parent R01 clinical trial Data Safety and Monitoring Board in the context of an ongoing Phase III clinical trial (ACT study, R01AG054077). All trial data will be made publicly available 2 years after completion of the parent clinical trial, per NIA and DSMB agreement. Requests to access these datasets should be directed to Adam J. Woods (adam.woods@utdallas.edu, ajwoods@phhp.ufl.edu).
