# Supplementary material for: Machine learning and individual variability in electrical field characteristics predict tDCS treatment response for anxiety in older adults in the ACT trial
Source: Front Hum Neurosci. 2026 Jul 31;20:1847131. doi: 10.3389/fnhum.2026.1847131 (PMC13473359; doi:10.3389/fnhum.2026.1847131)

Temporal Fusiform Cortex, posterior division

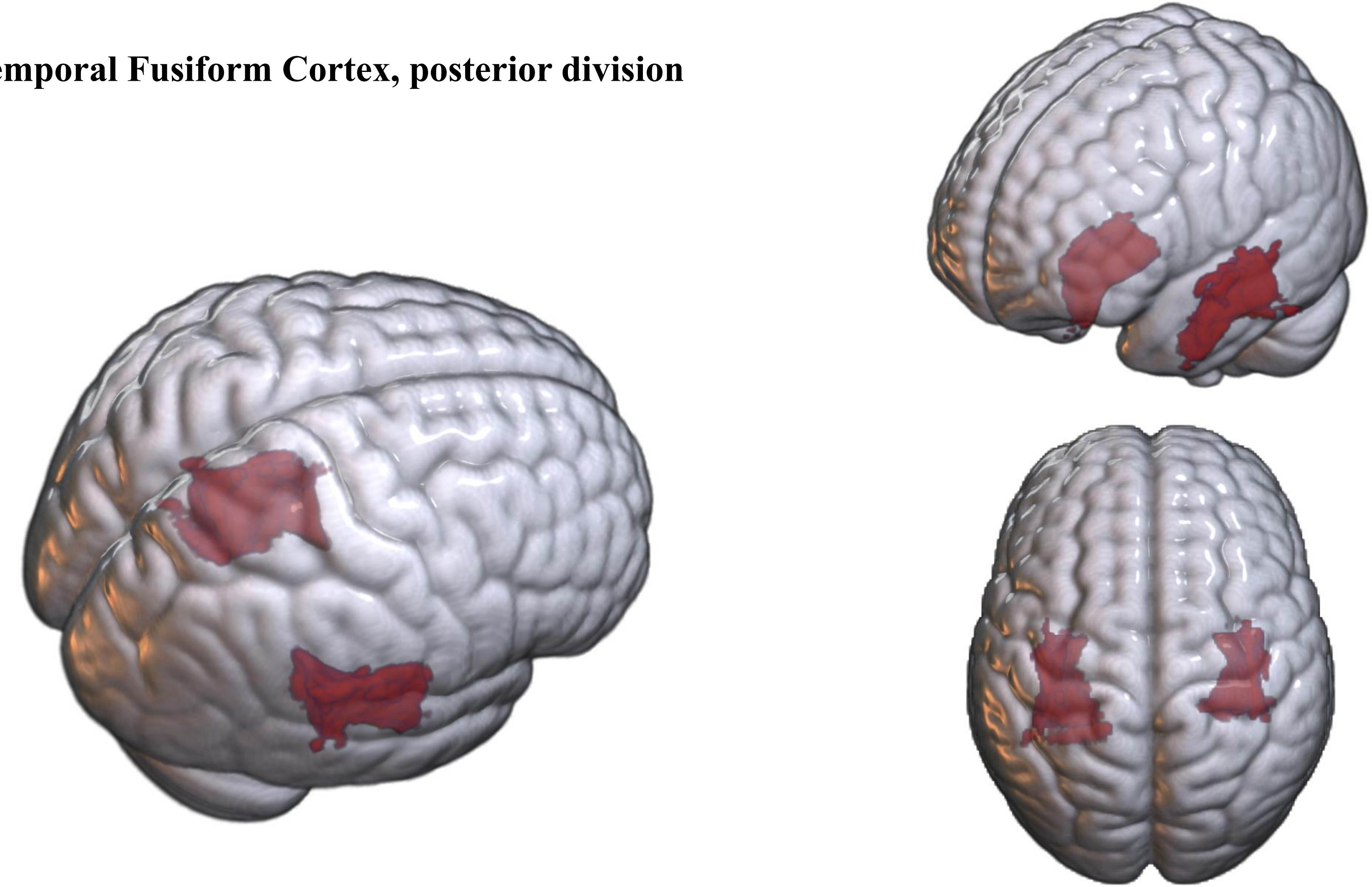

Greater-improvement Participant in Training Set

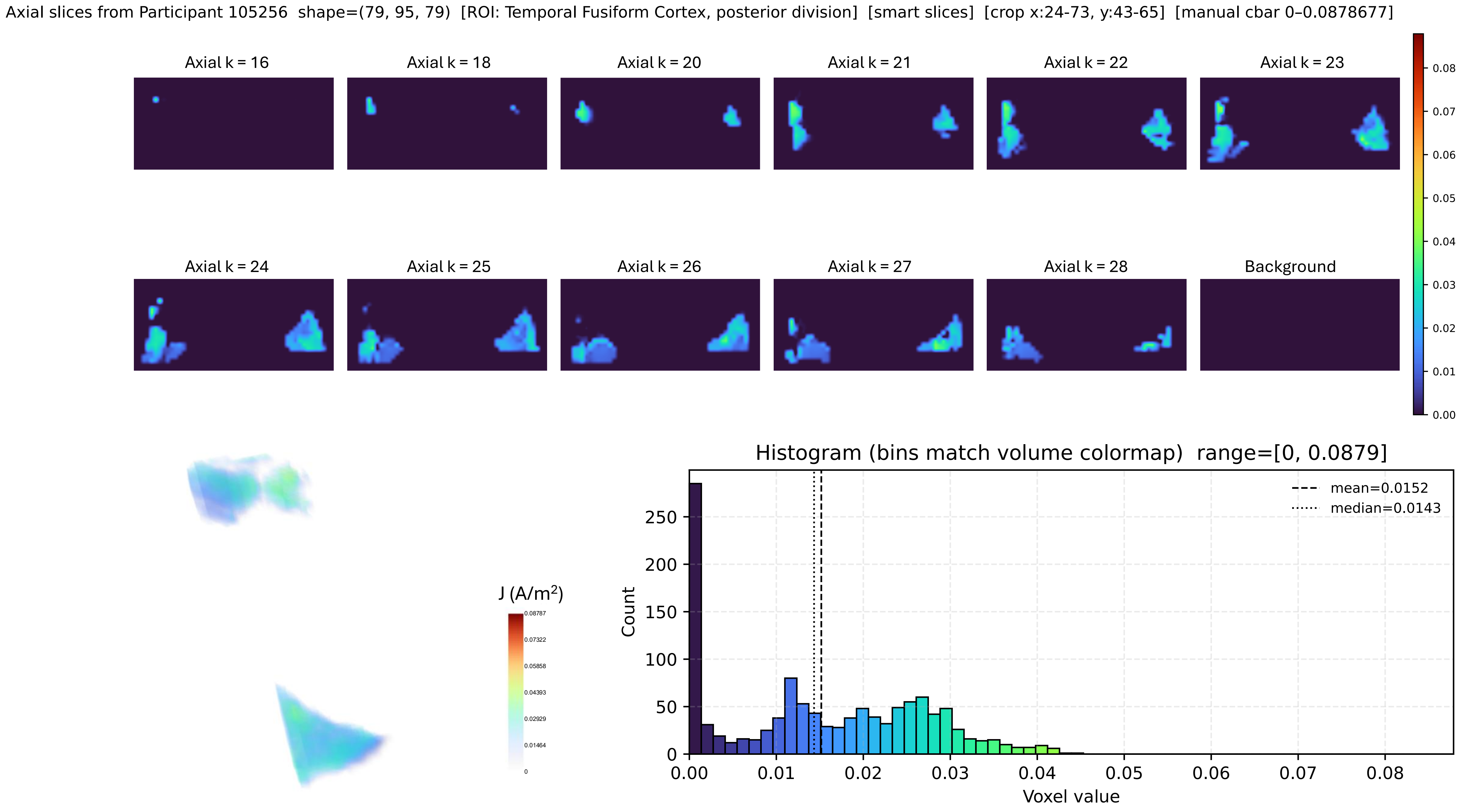

Greater-improvement Participant in Training Set

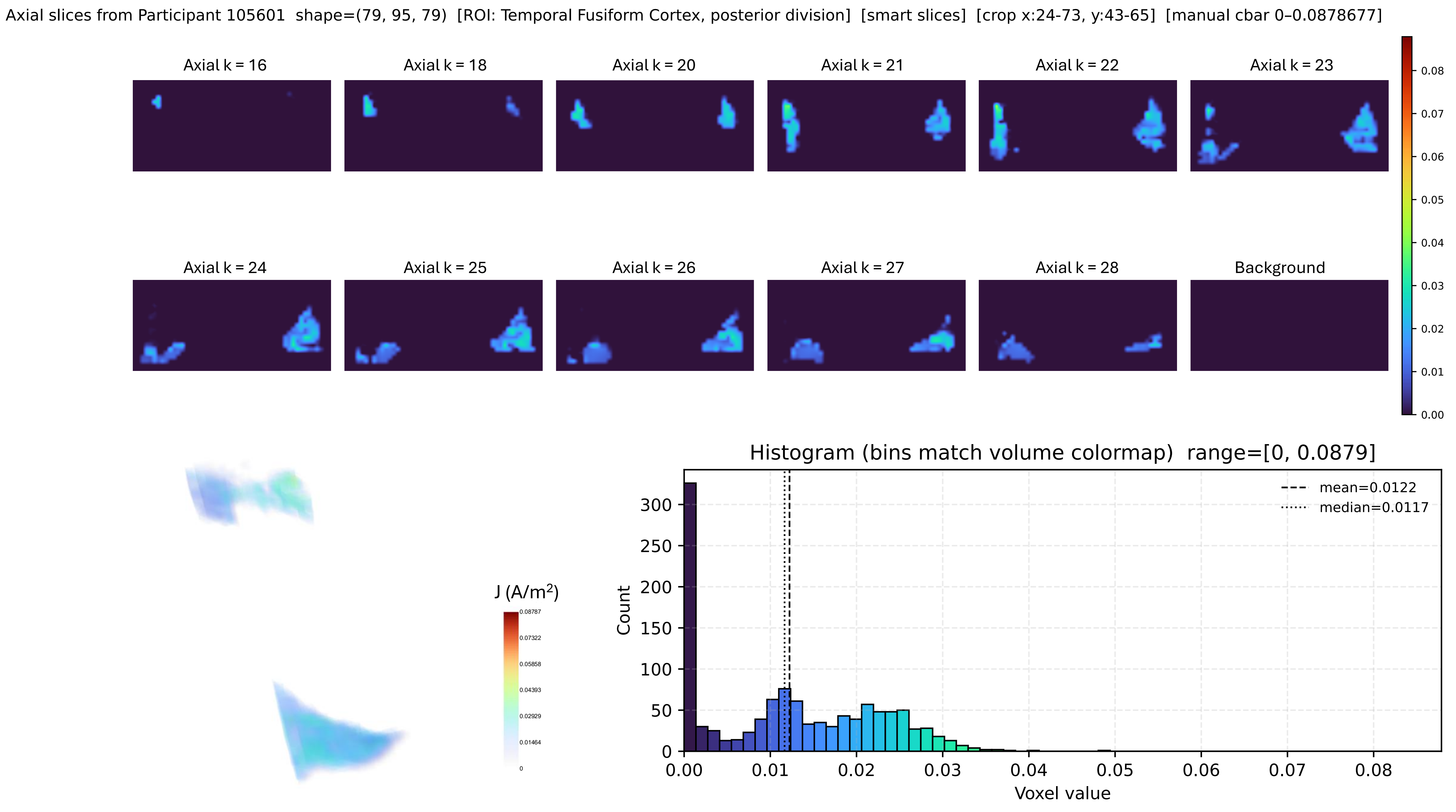

Greater-improvement Participant in Training Set

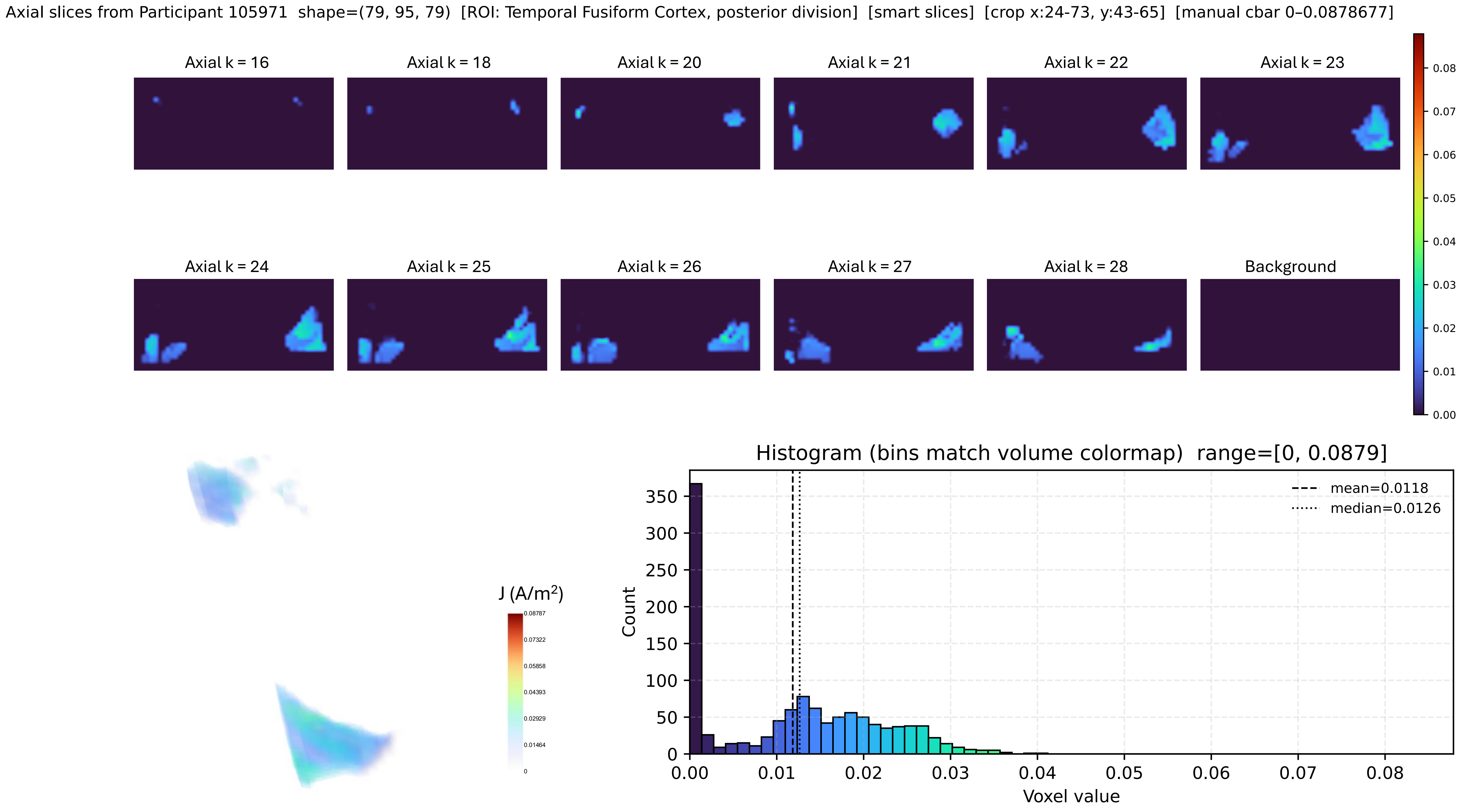

Greater-improvement Participant in Training Set

Axial slices from Participant 107802 shape=(79, 95, 79) [ROI: Temporal Fusiform Cortex, posterior division] [smart slices] [crop x:24-73, y:43-65] [manual cbar 0–0.0878677]

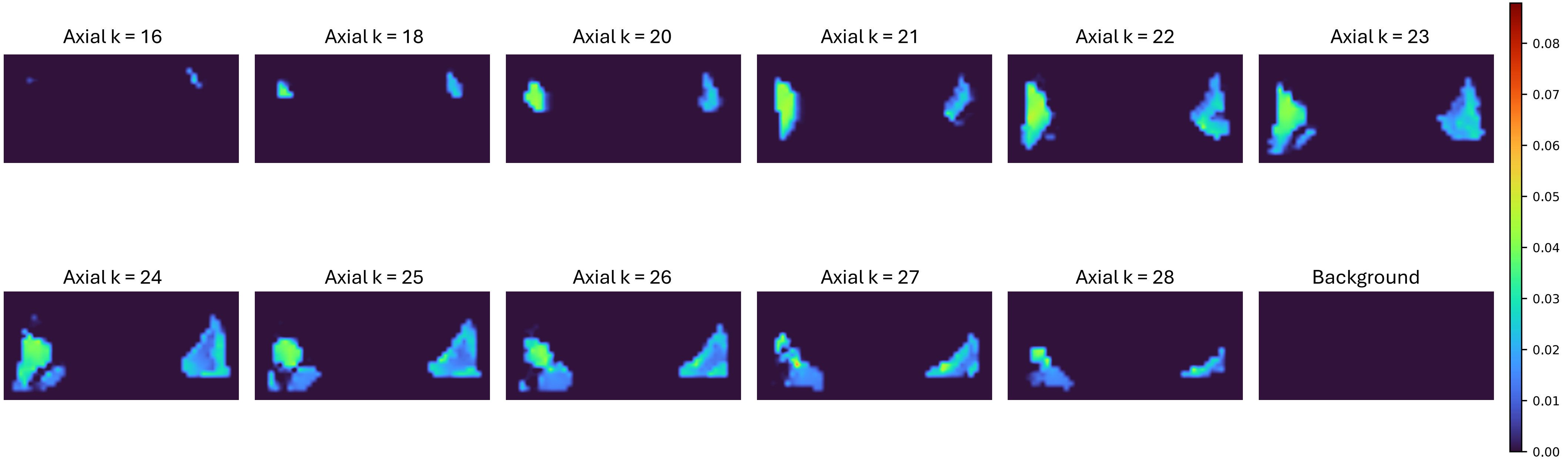

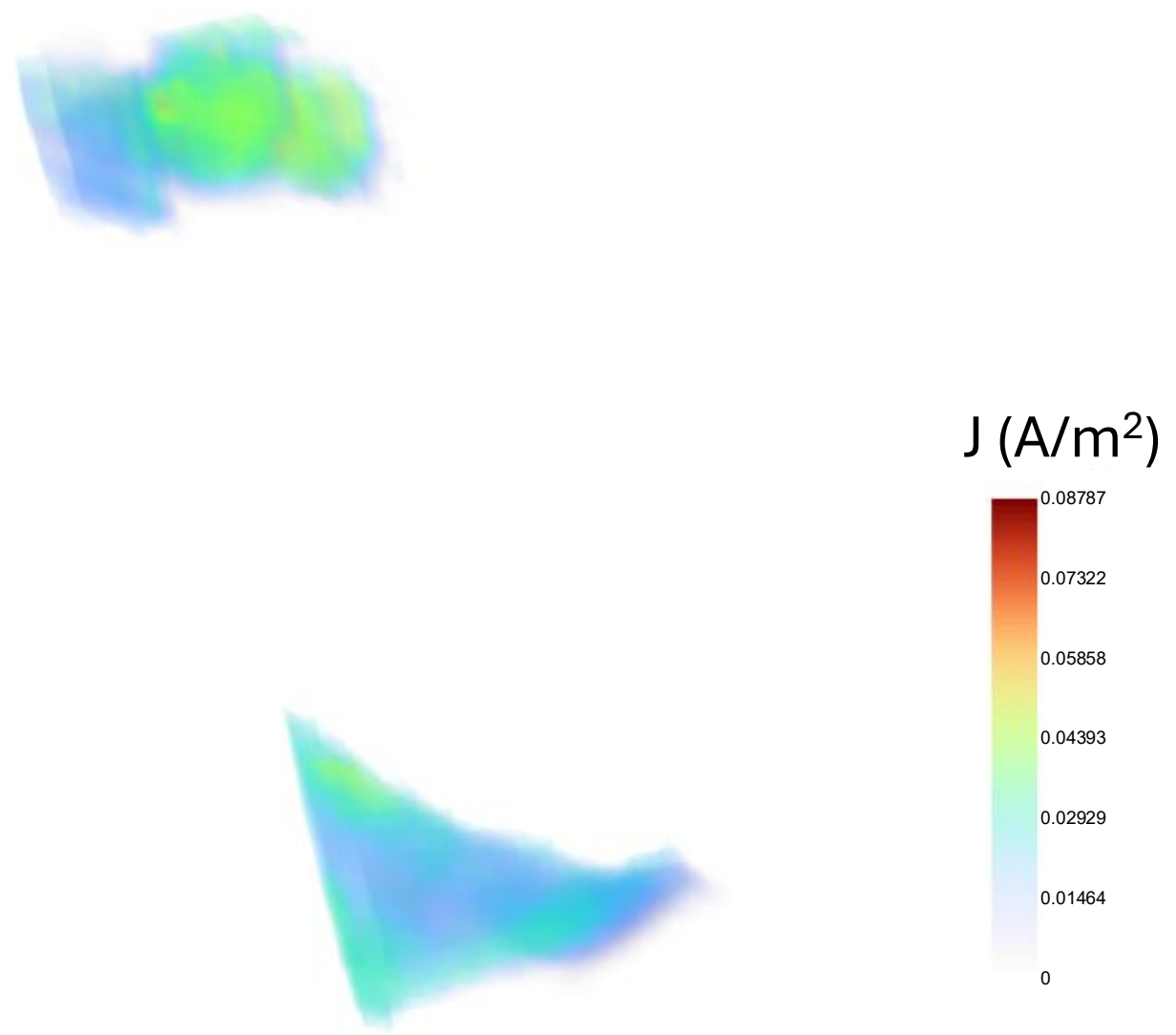

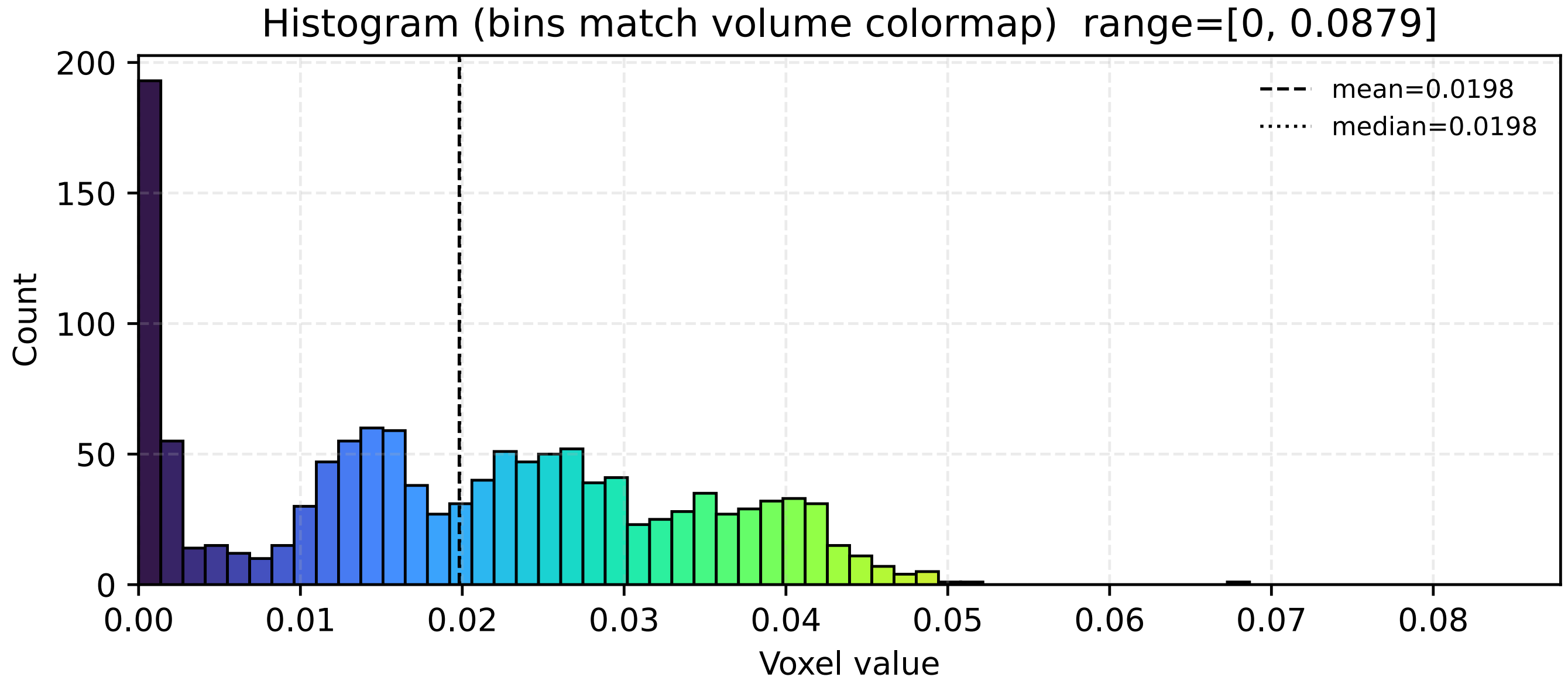

Greater-improvement Participant in Training Set

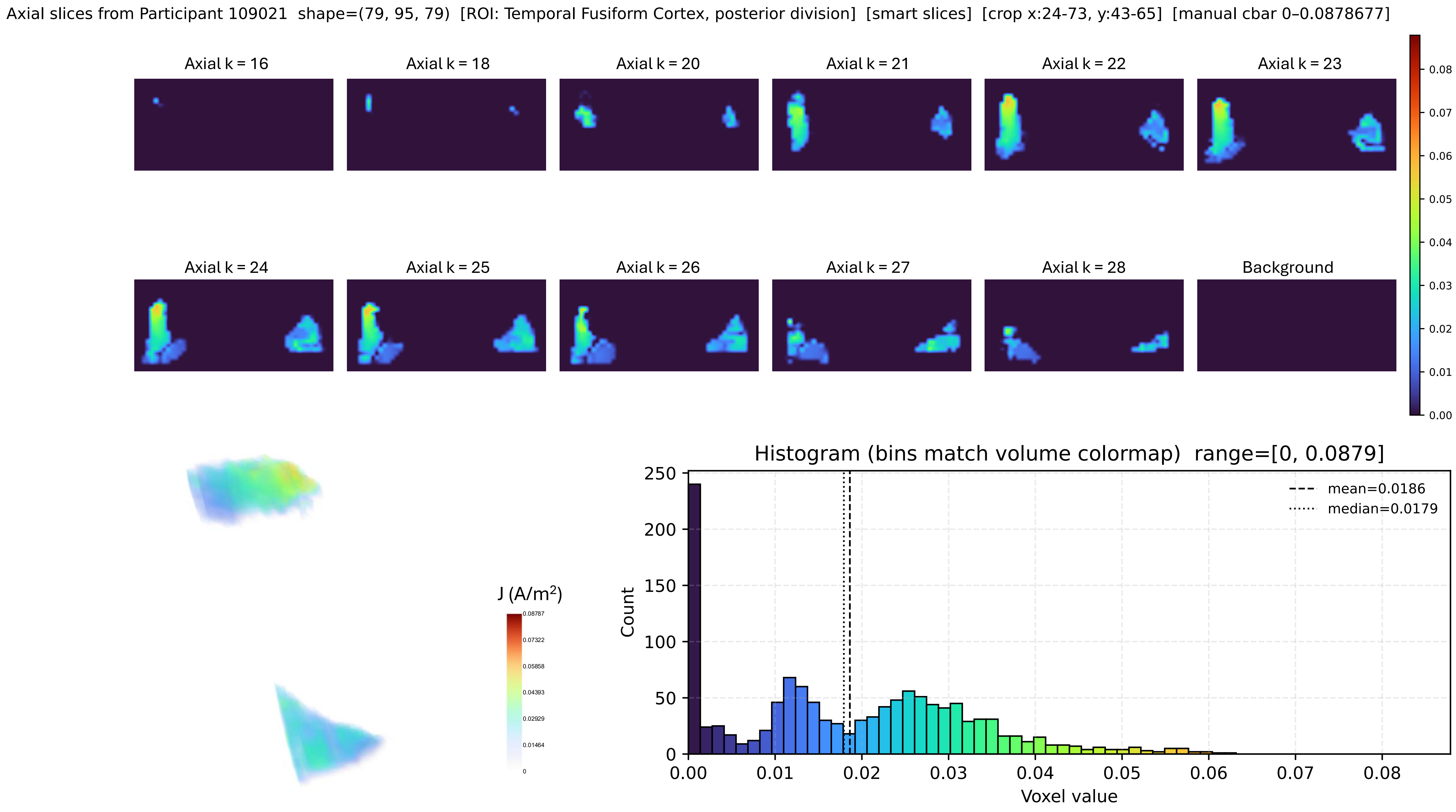

Greater-improvement Participant in Training Set

Axial slices from Participant 202808 shape=(79, 95, 79) [ROI: Temporal Fusiform Cortex, posterior division] [smart slices] [crop x:24-73, y:43-65] [manual cbar 0–0.0878677]

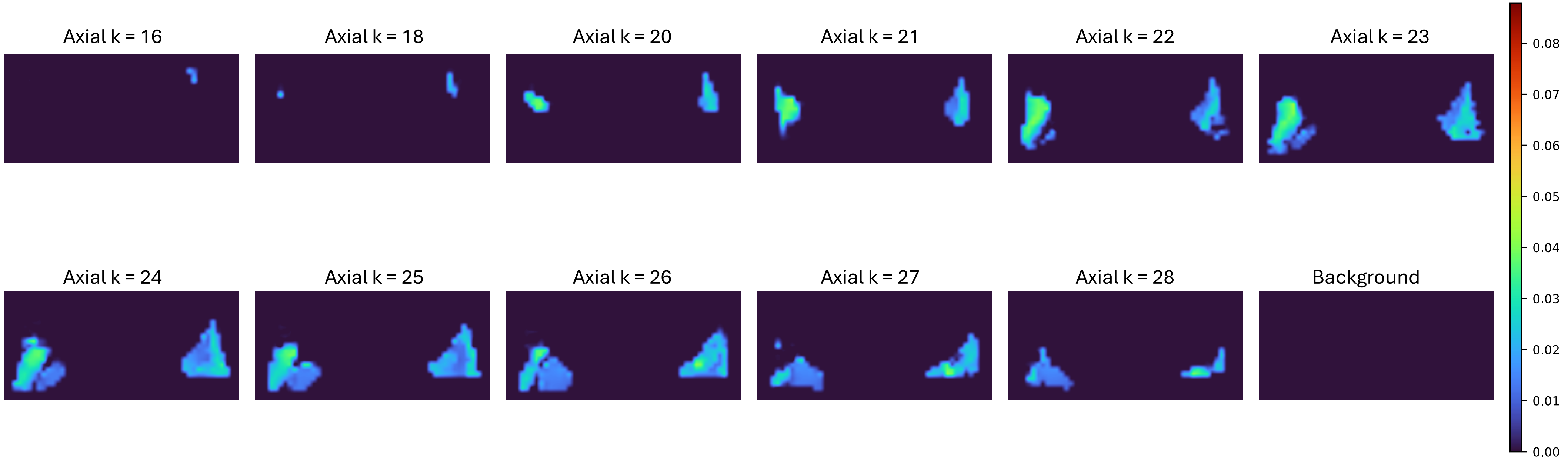

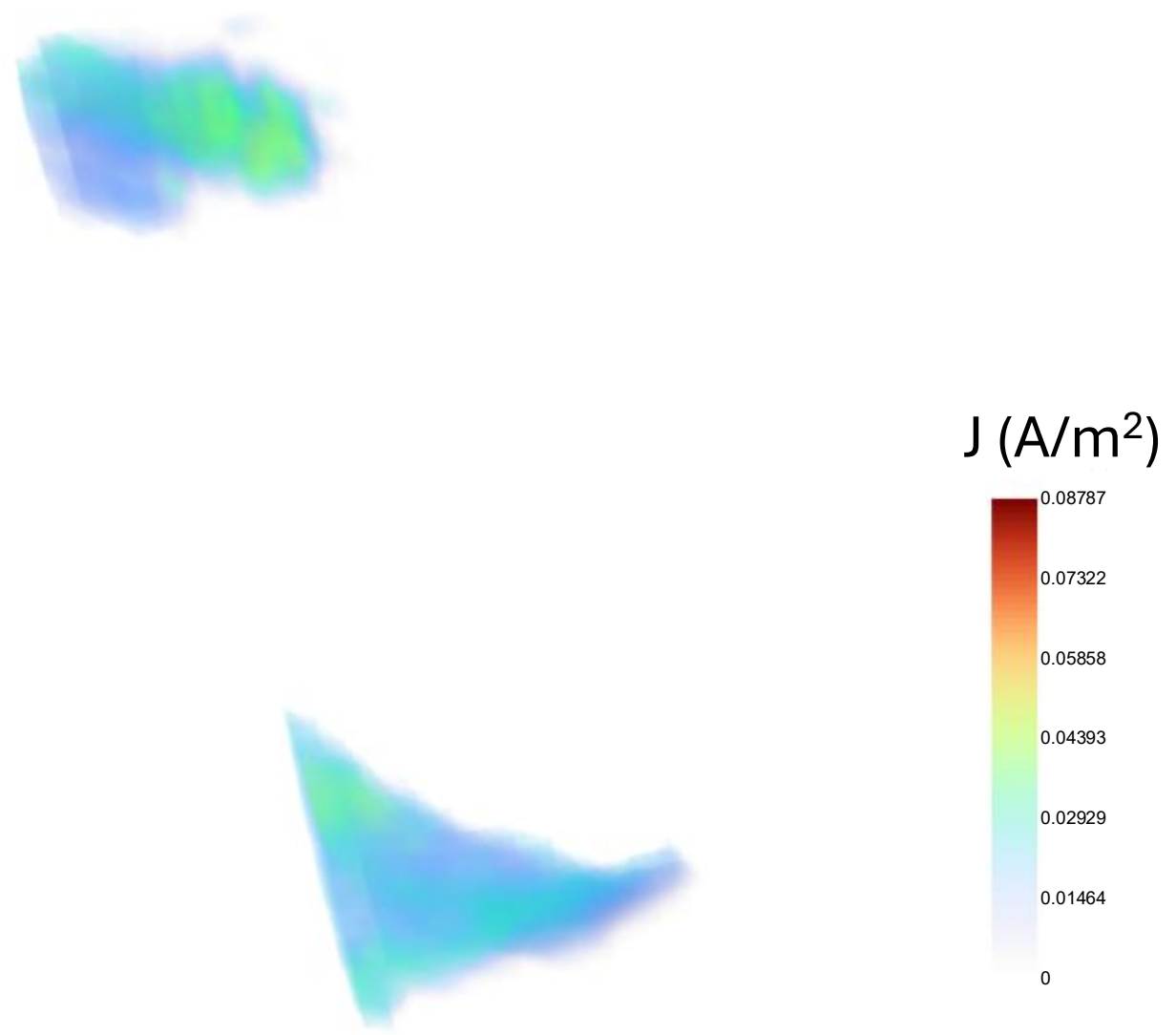

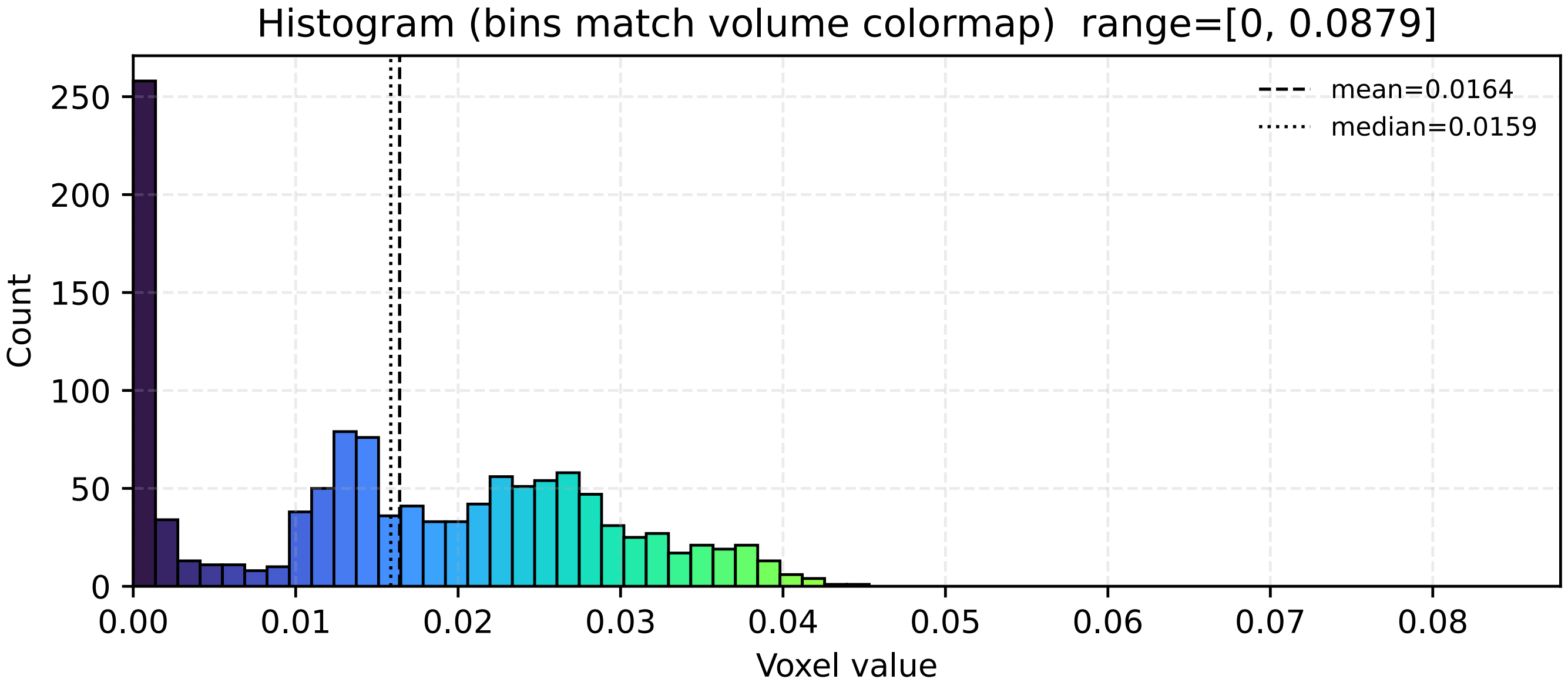

Greater-improvement Participant in Training Set

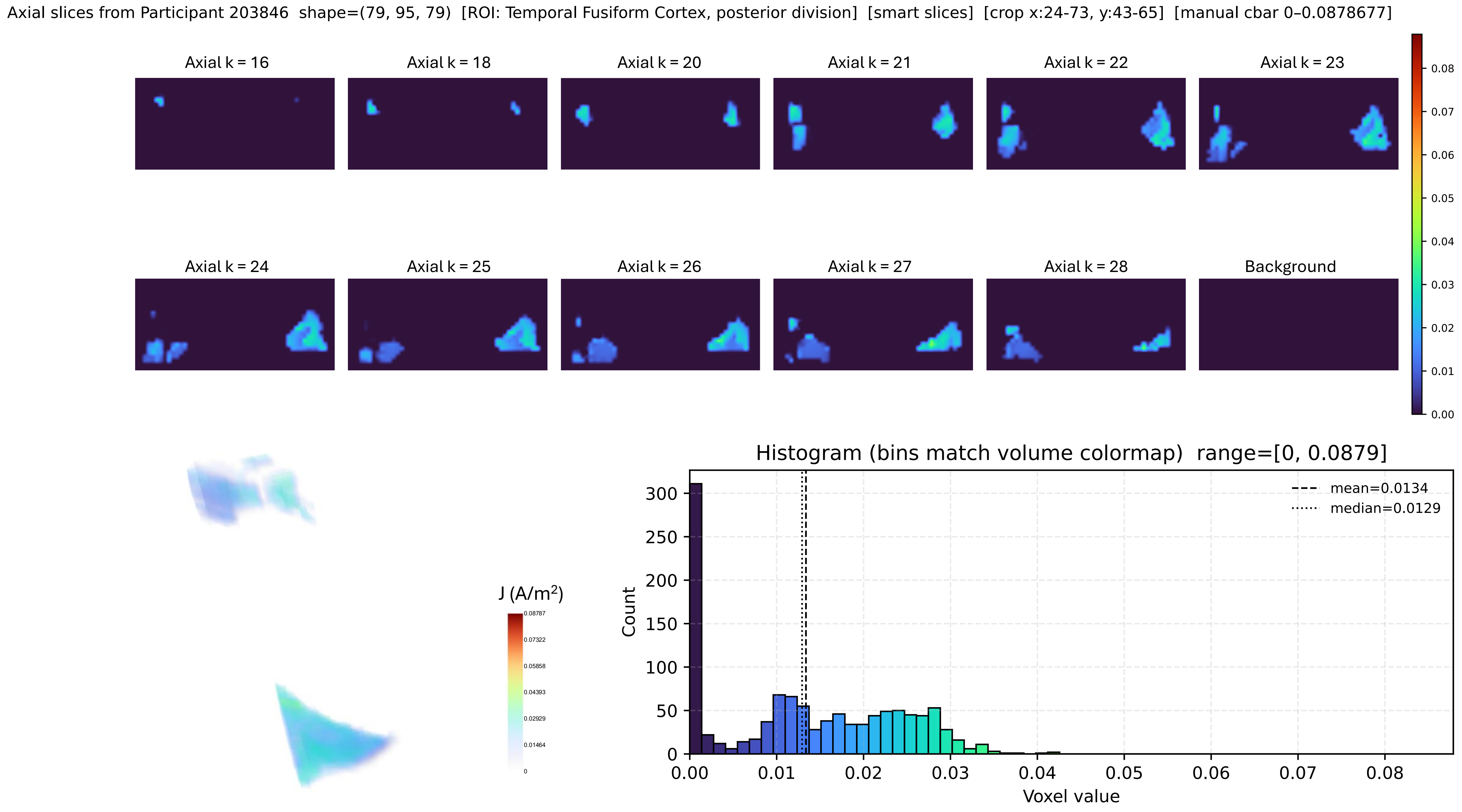

Lesser-improvement Participant in Training Set

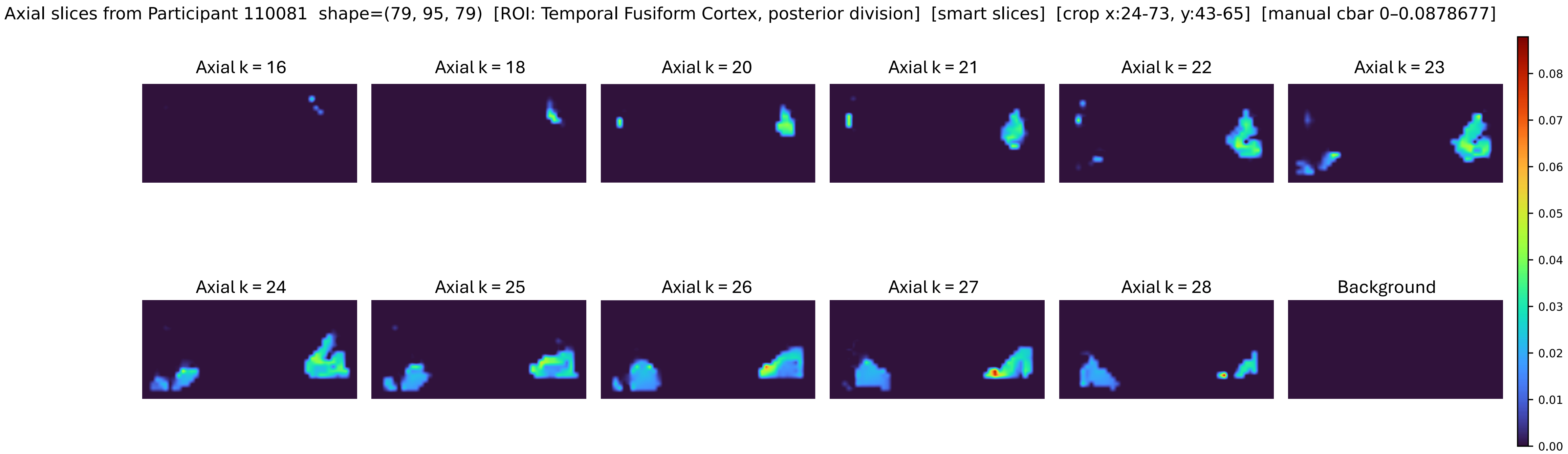

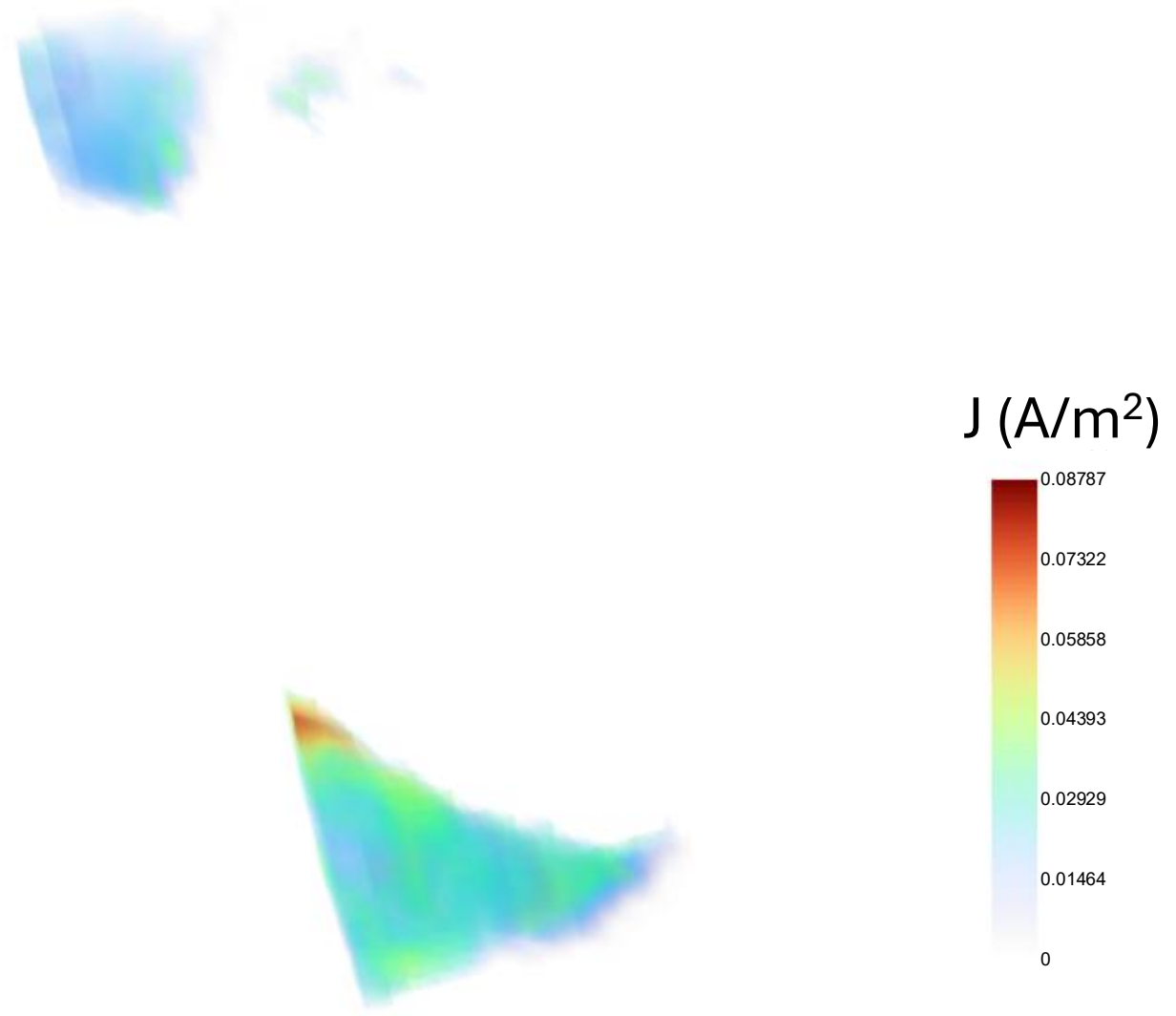

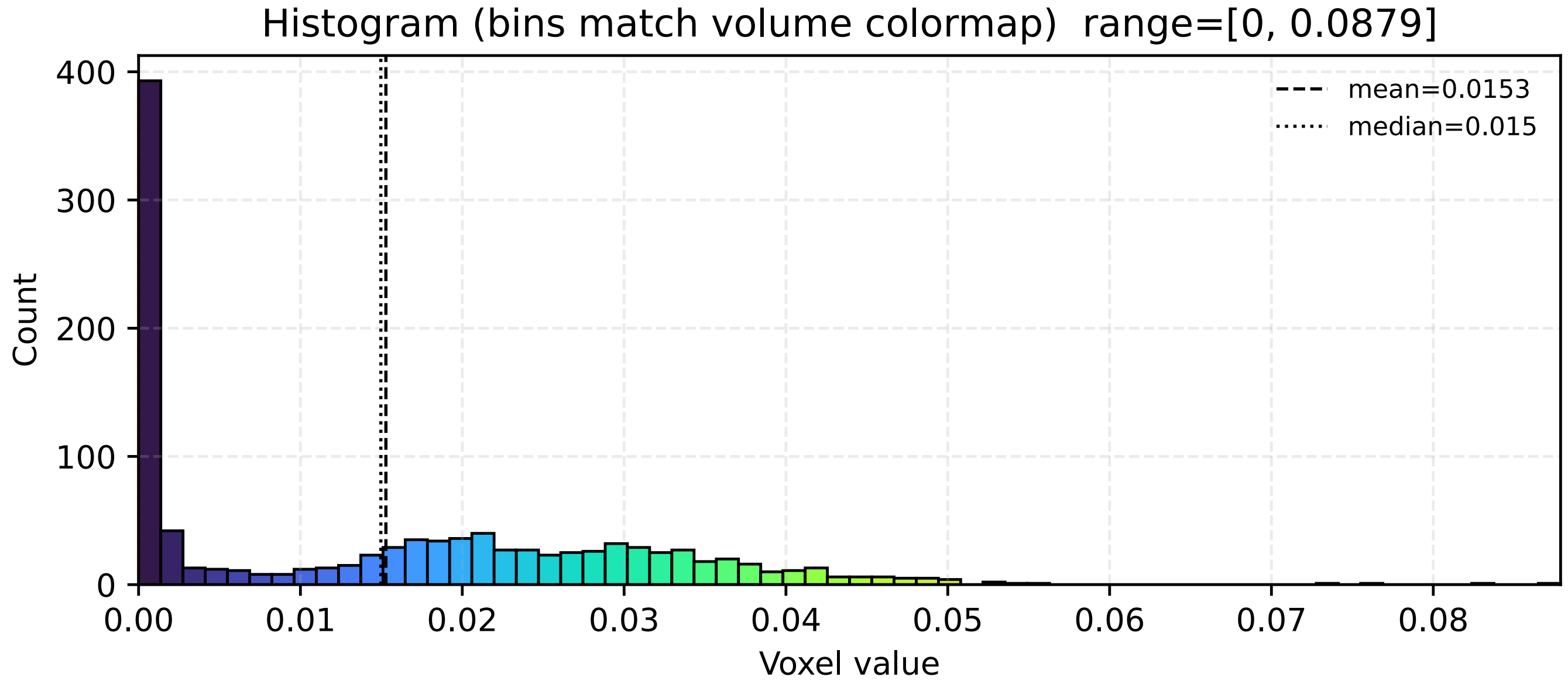

Lesser-improvement Participant in Training Set

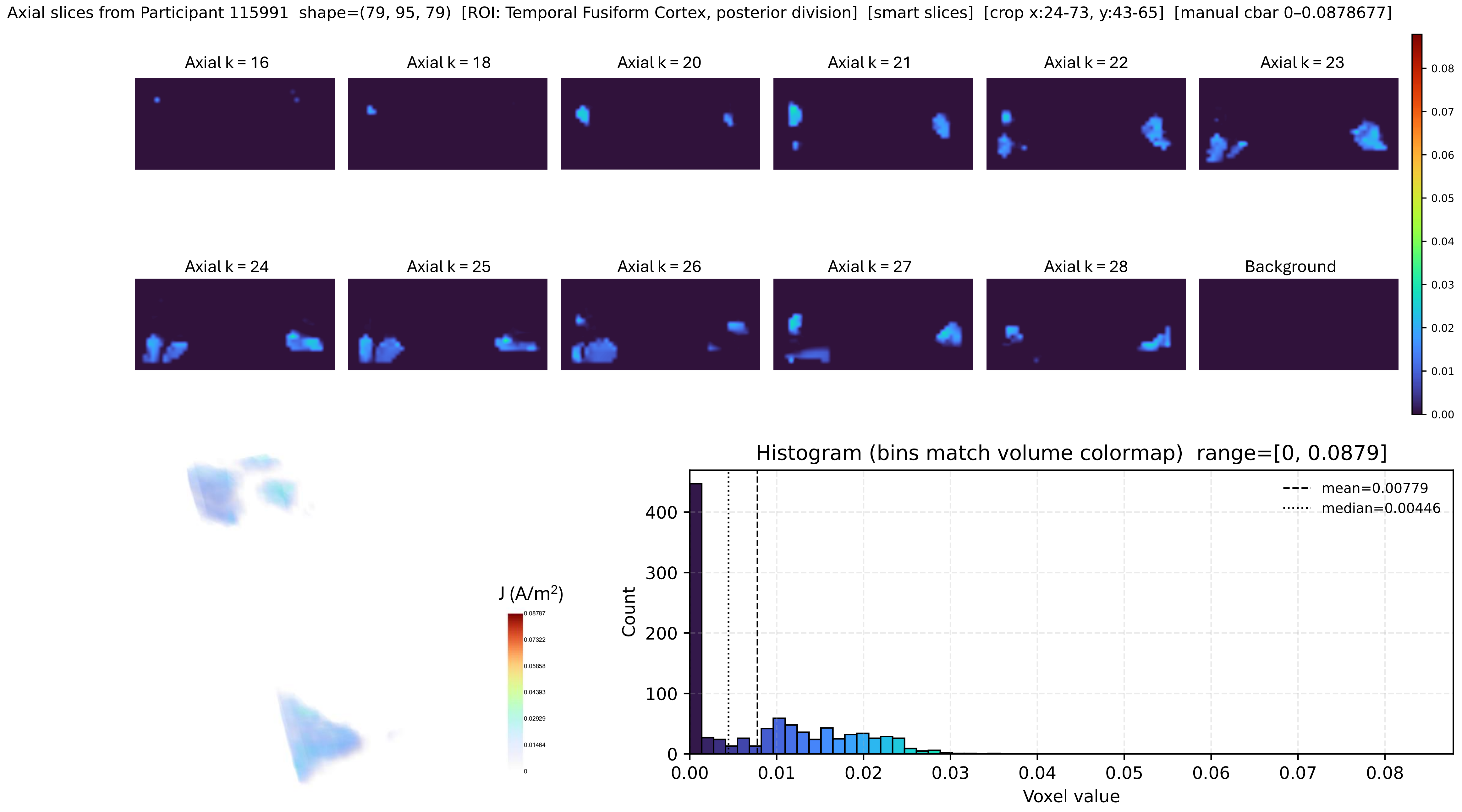

Lesser-improvement Participant in Training Set

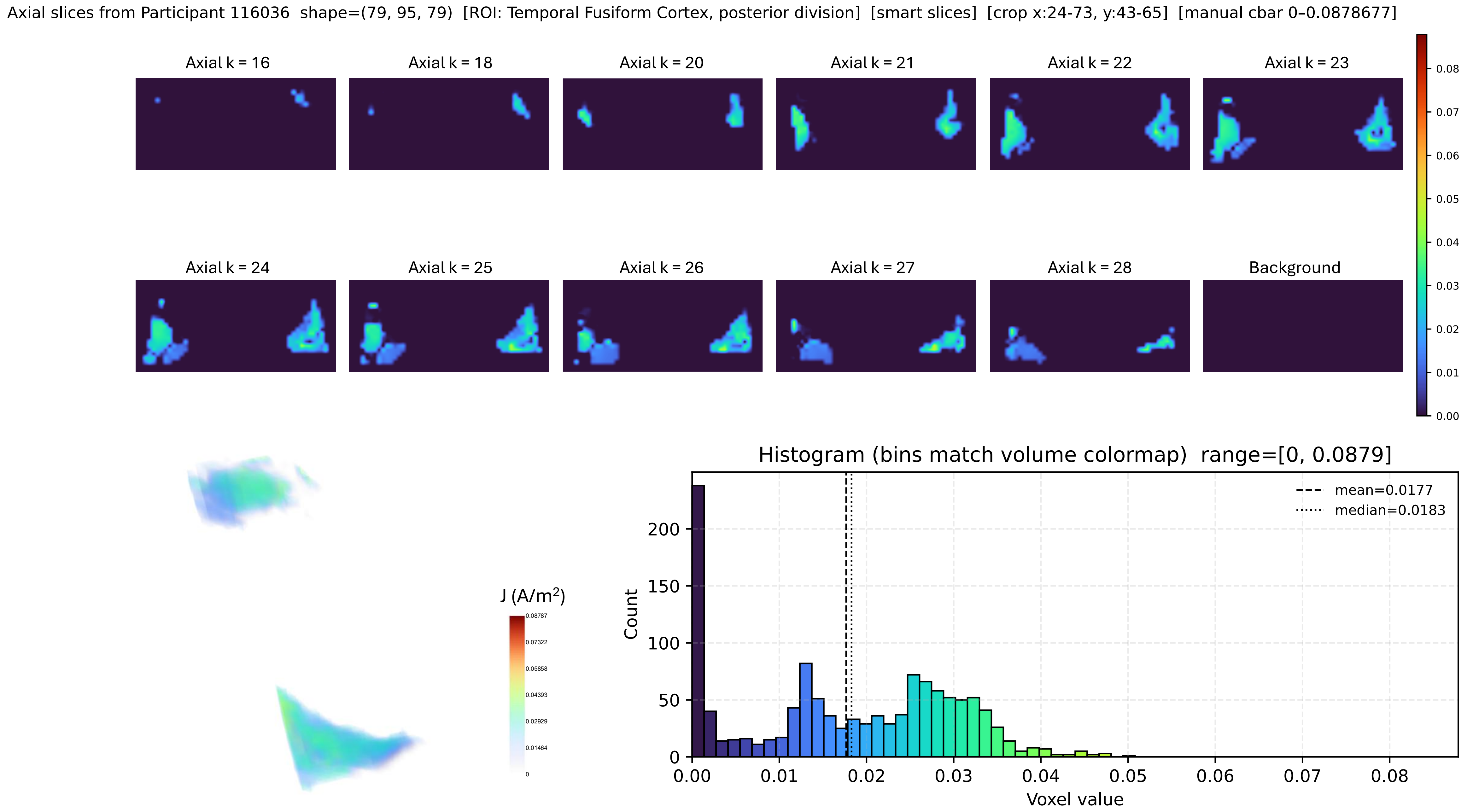

Lesser-improvement Participant in Training Set

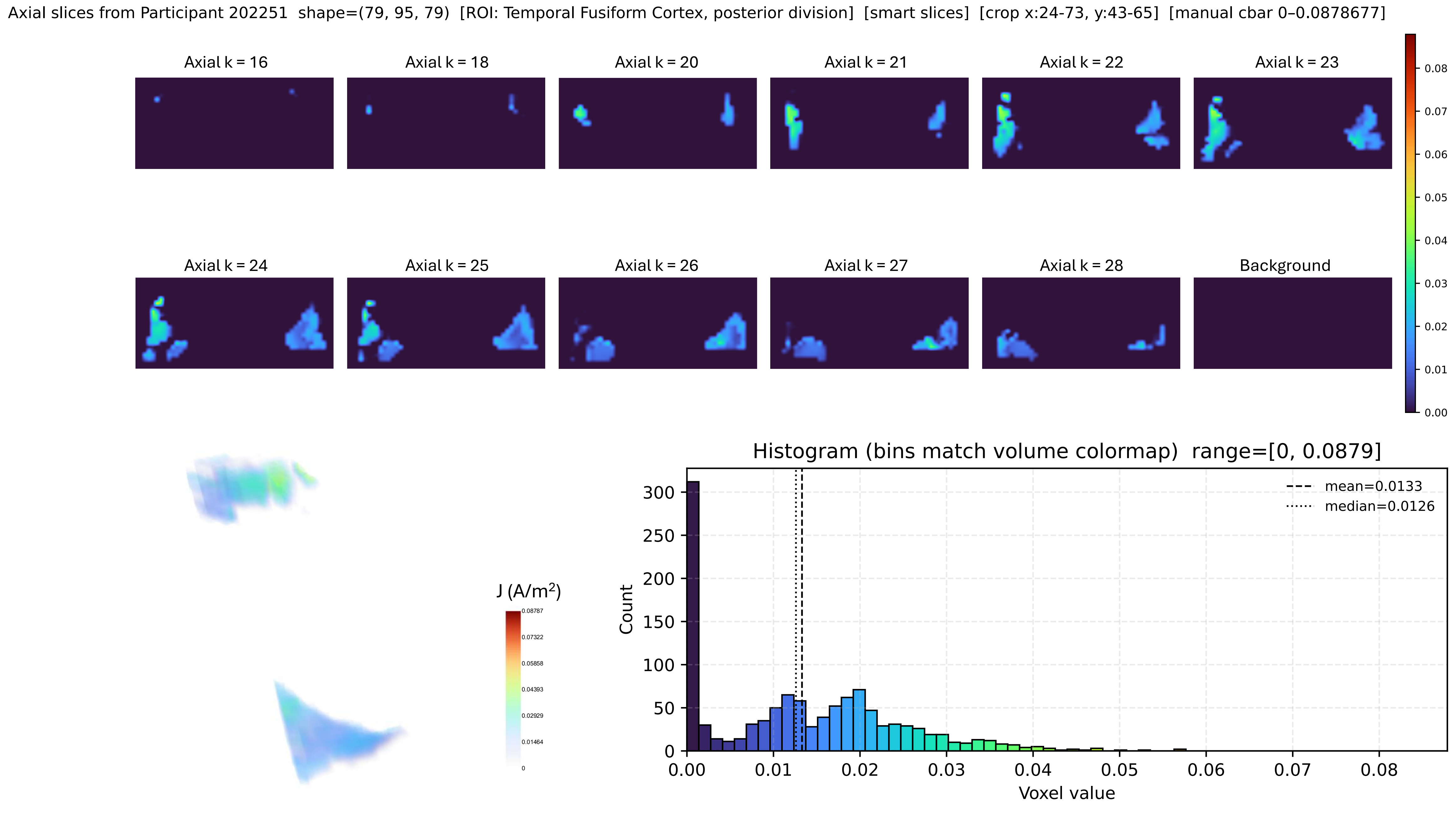

Greater-improvement Participant in Testing Set

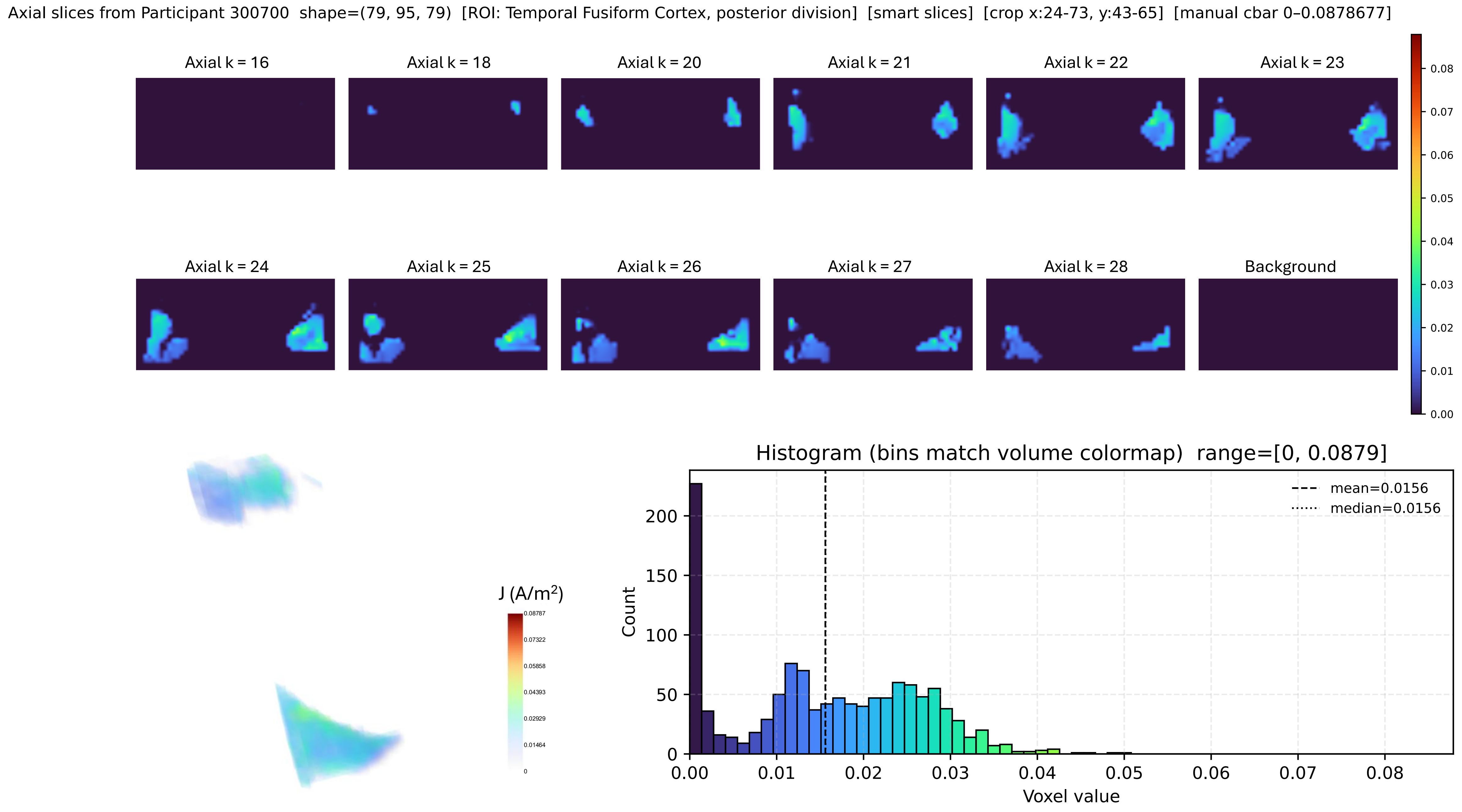

Greater-improvement Participant in Testing Set

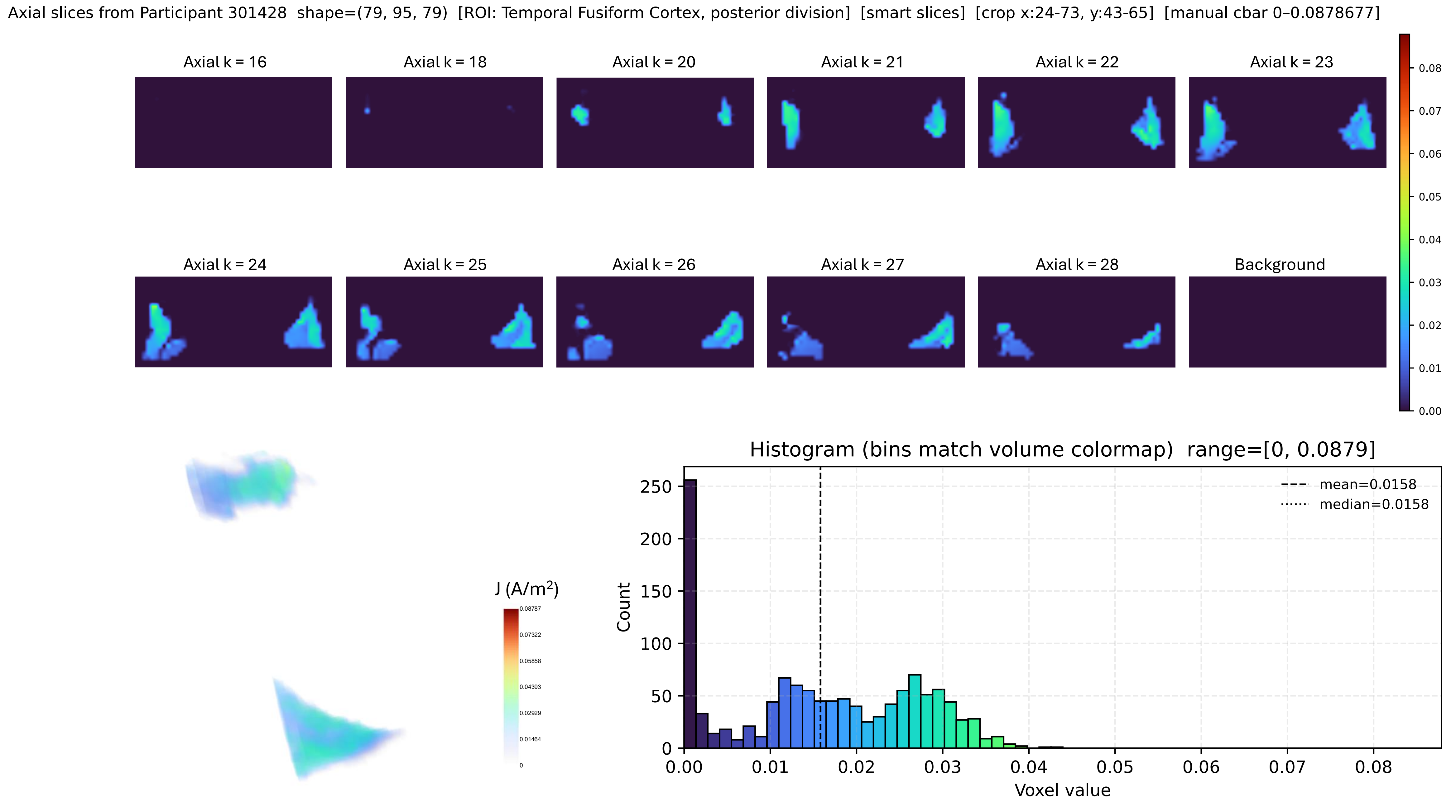

Greater-improvement Participant in Testing Set

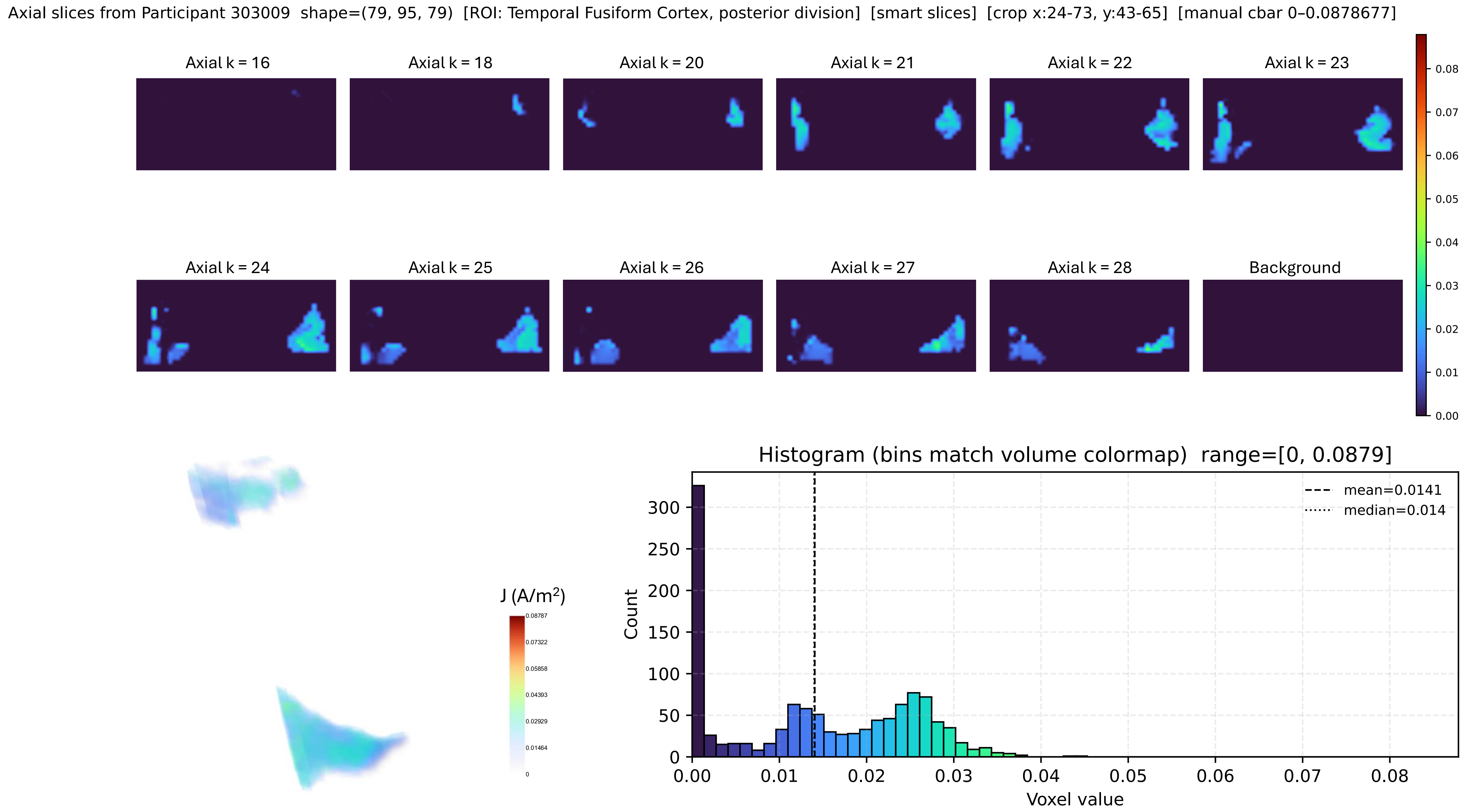

Greater-improvement Participant in Testing Set

Axial slices from Participant 303346 shape=(79, 95, 79) [ROI: Temporal Fusiform Cortex, posterior division] [smart slices] [crop x:24-73, y:43-65] [manual cbar 0–0.0878677]

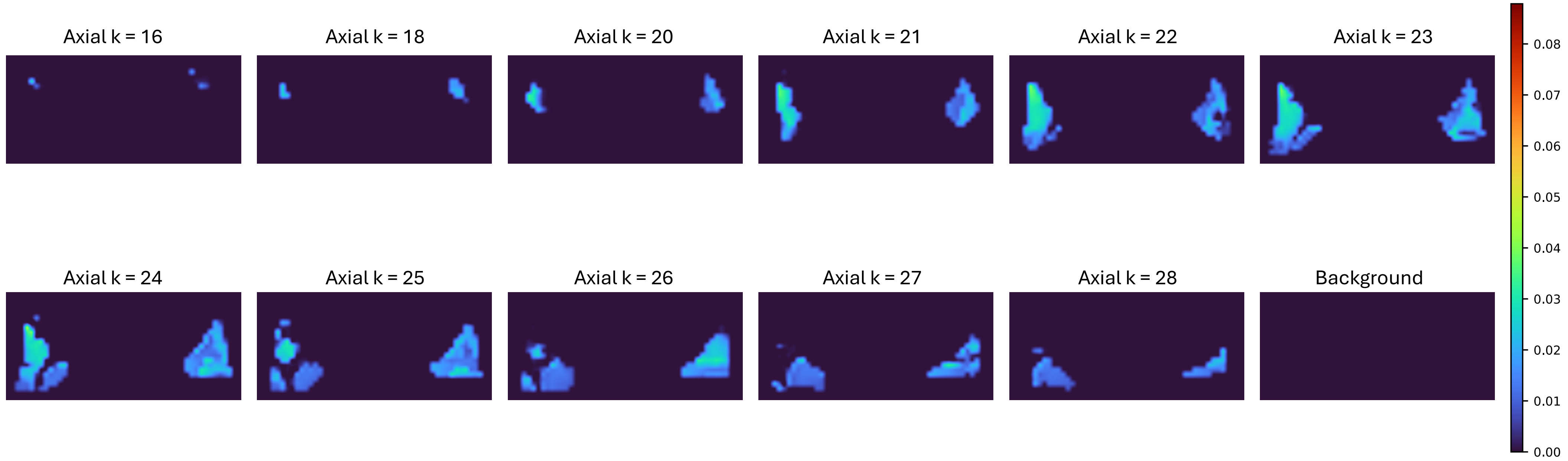

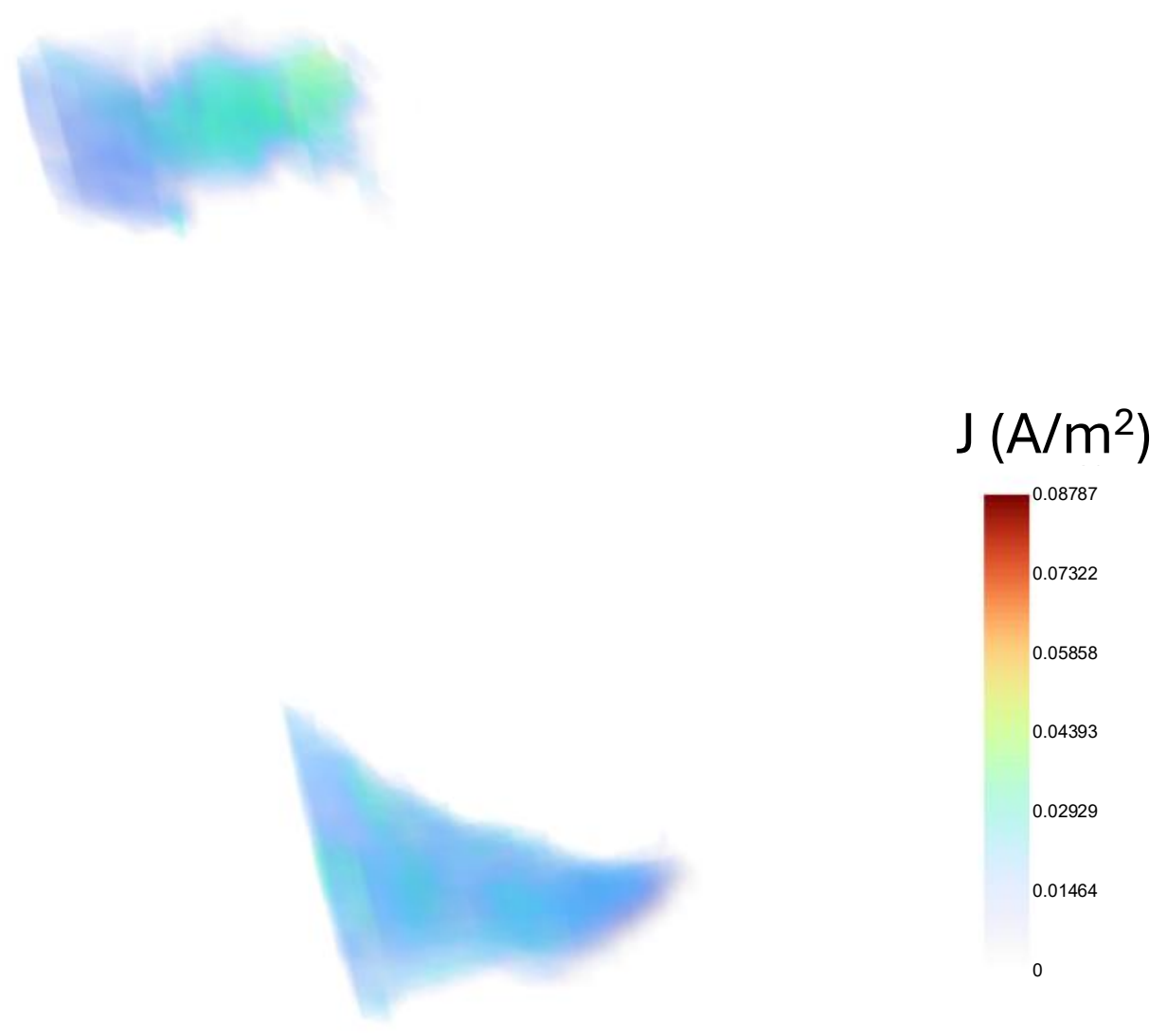

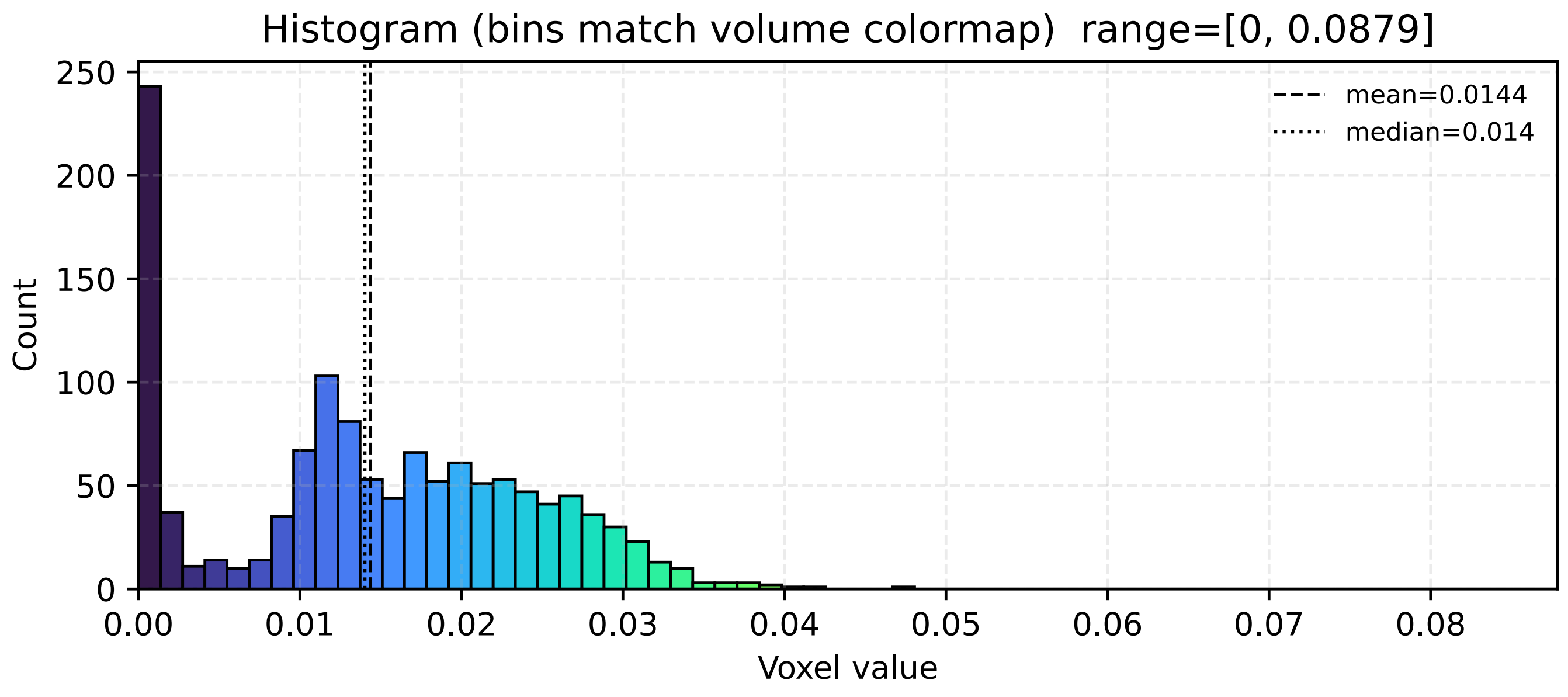

Lesser-improvement Participant in Testing Set

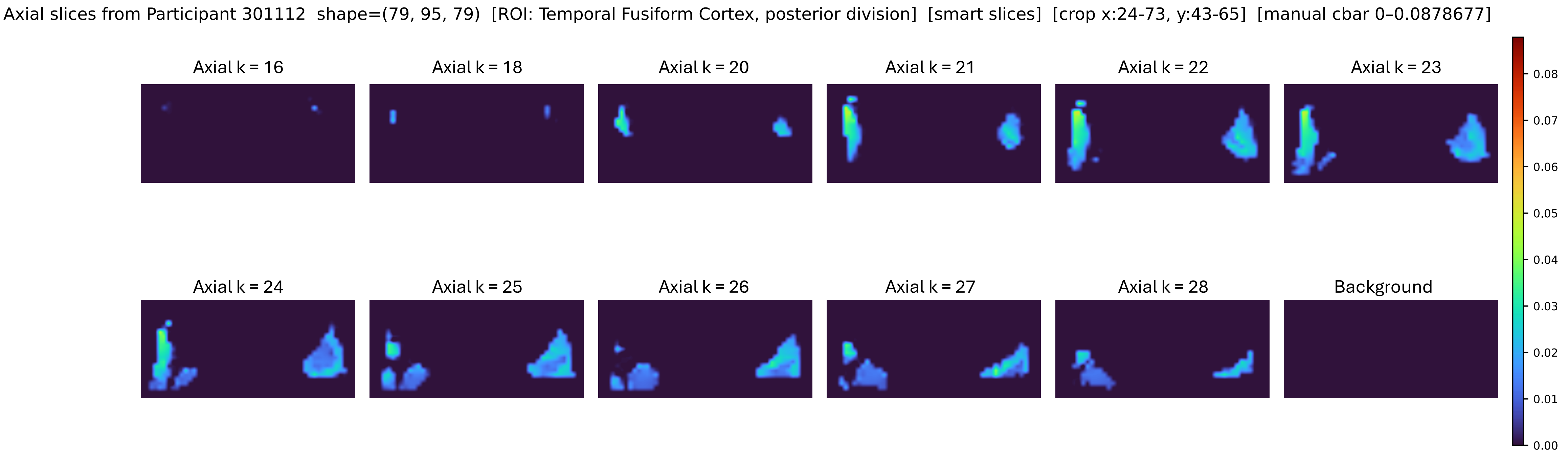

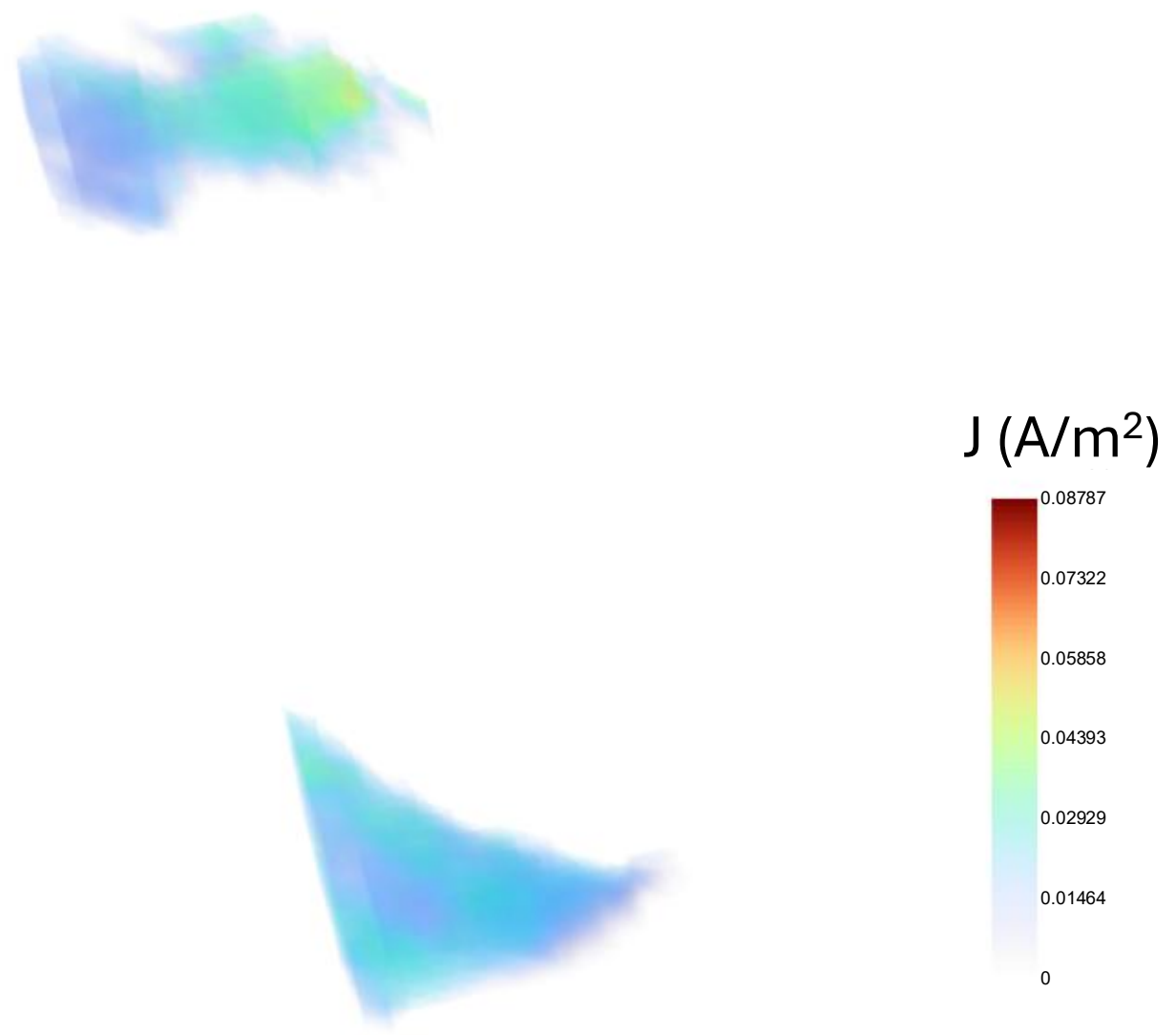

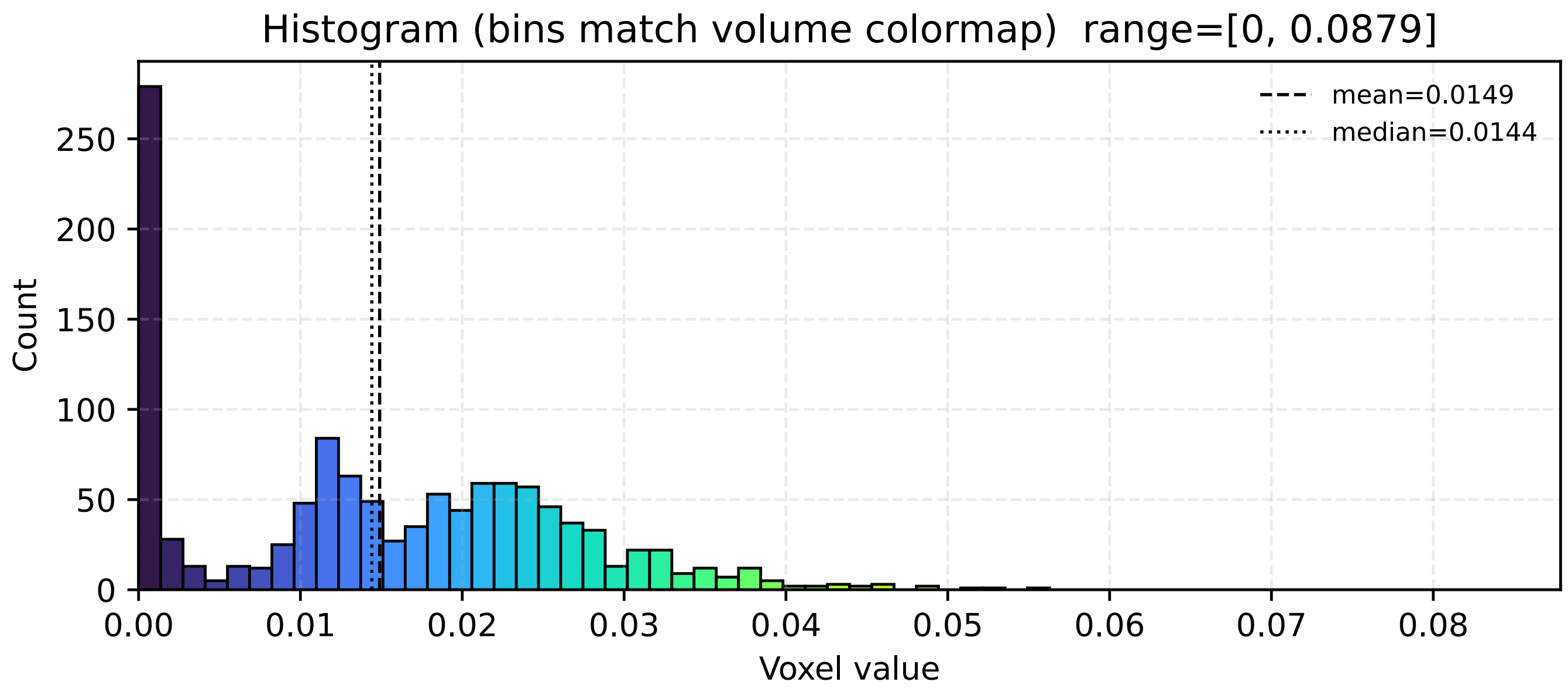

Lesser-improvement Participant in Testing Set

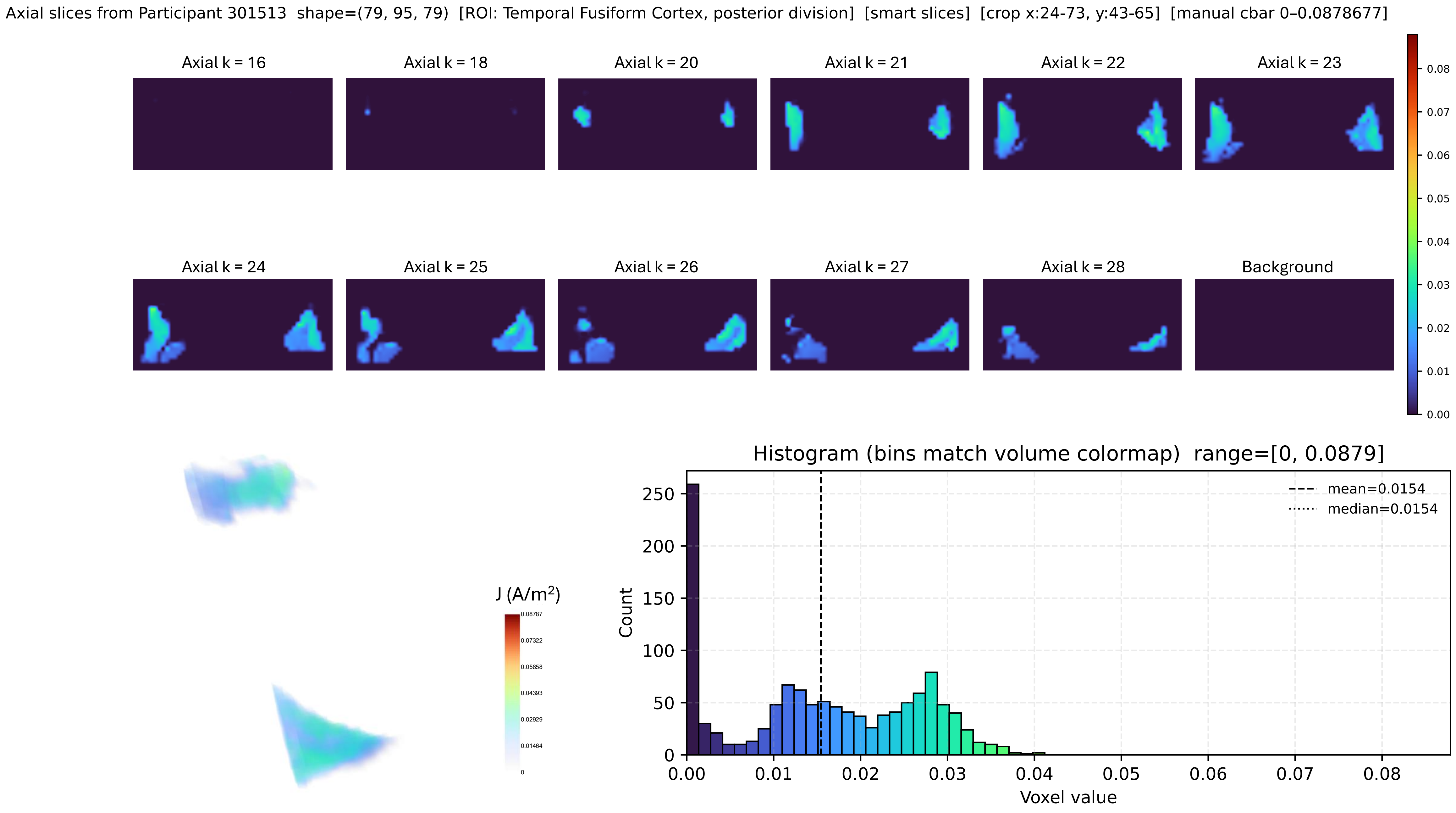

Lesser-improvement Participant in Testing Set

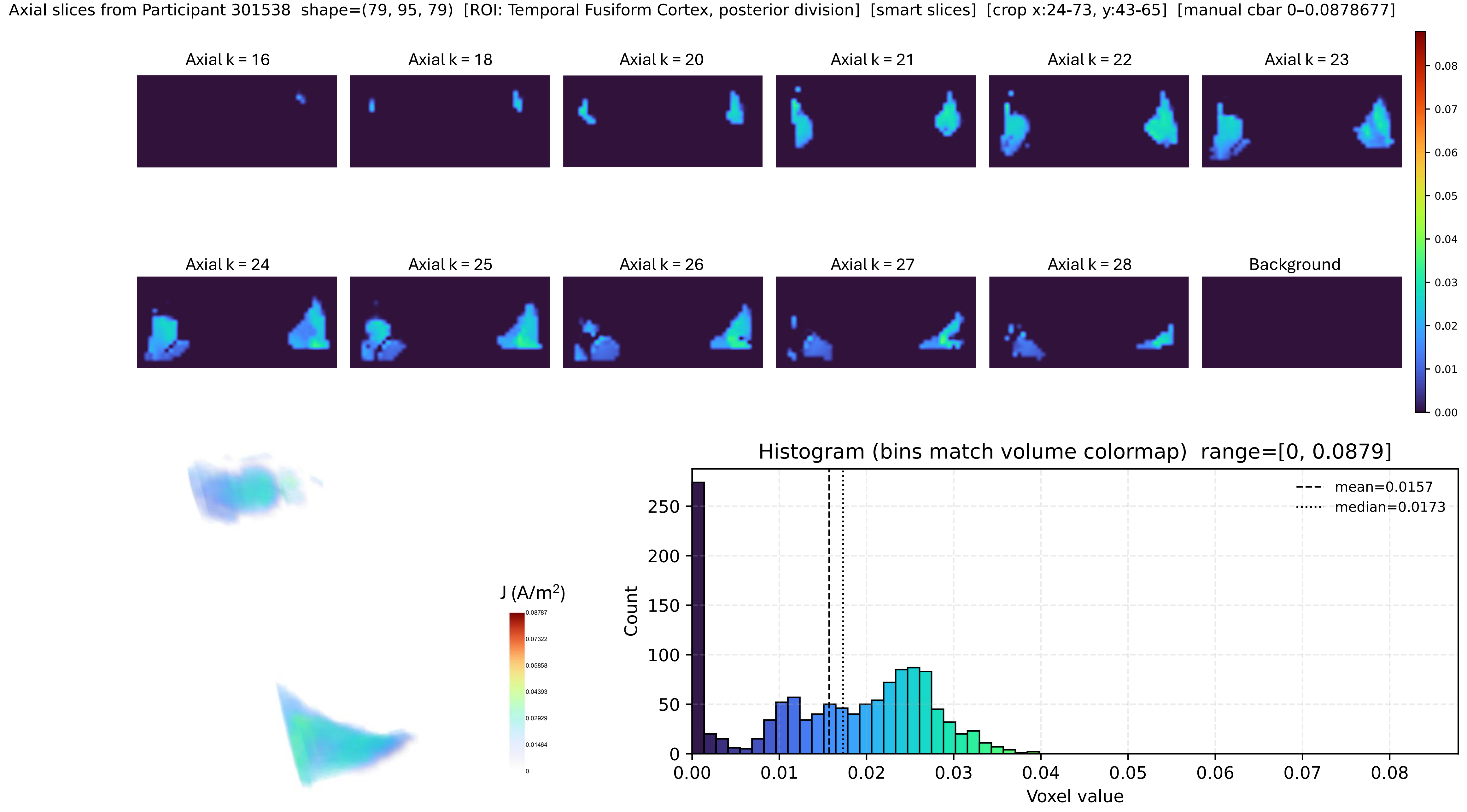

Lesser-improvement Participant in Testing Set

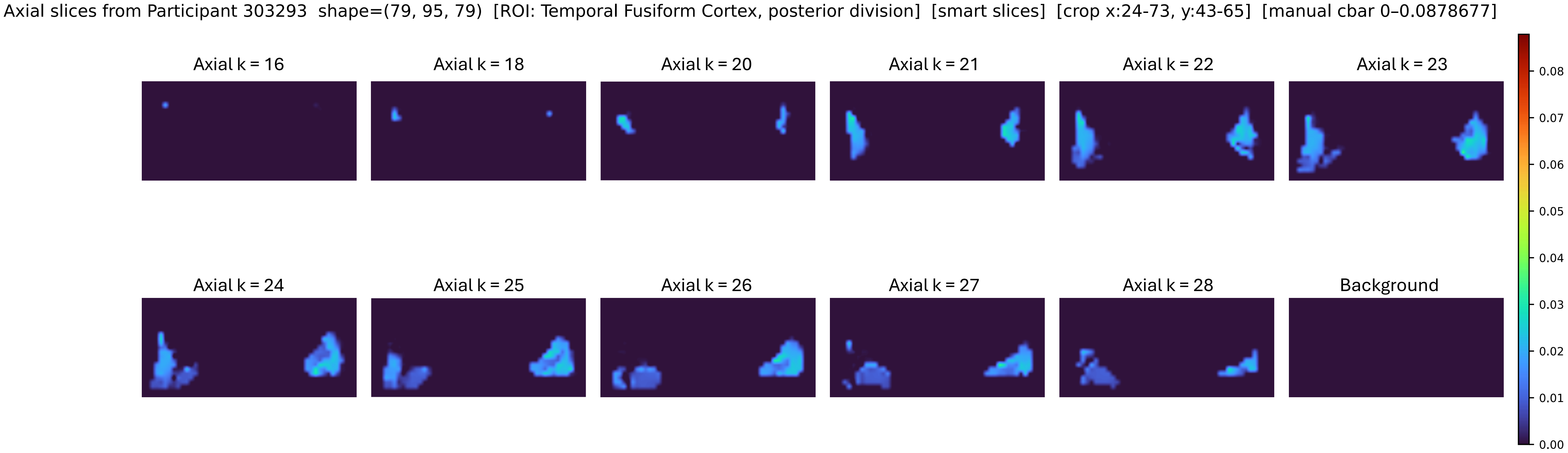

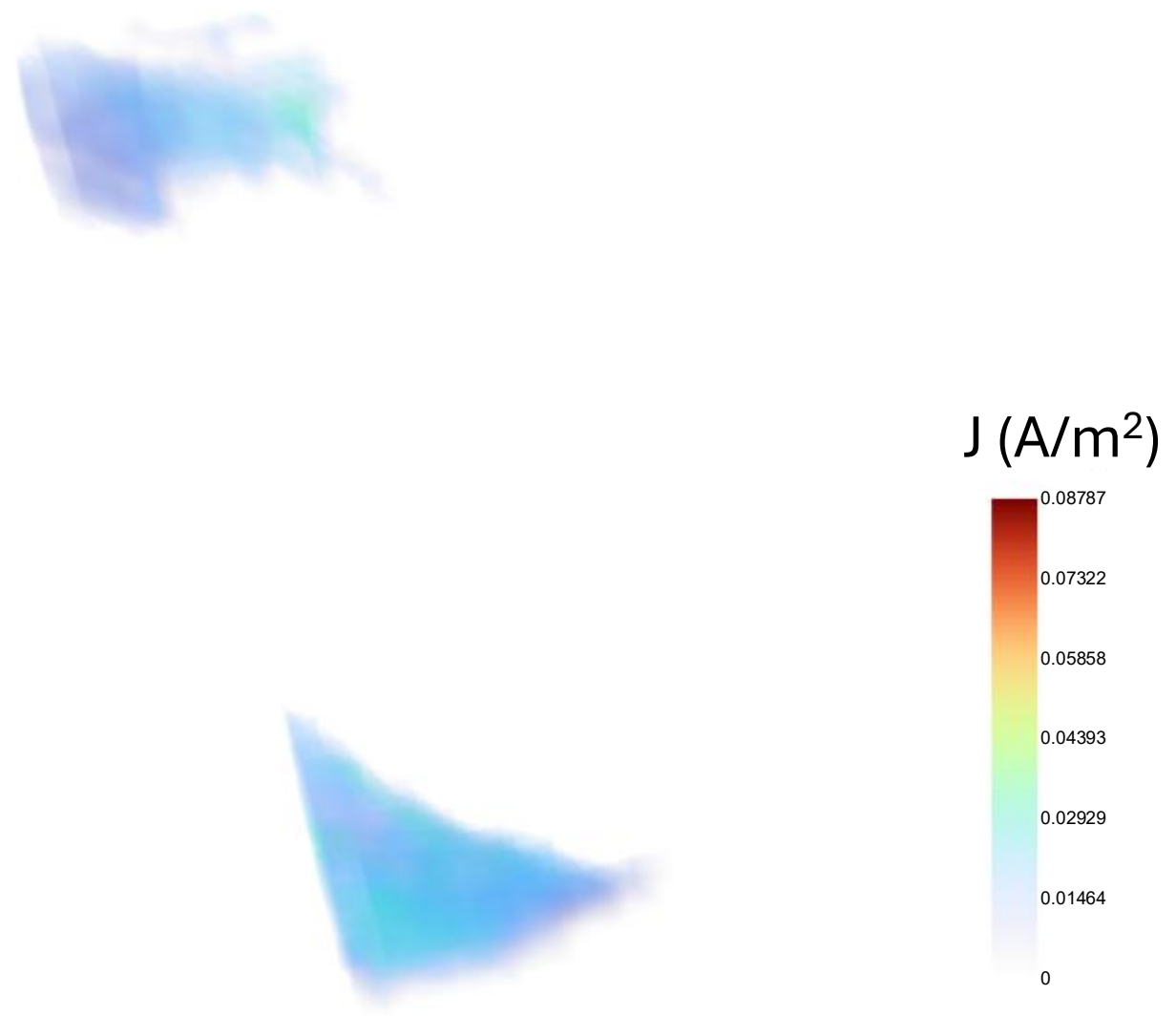

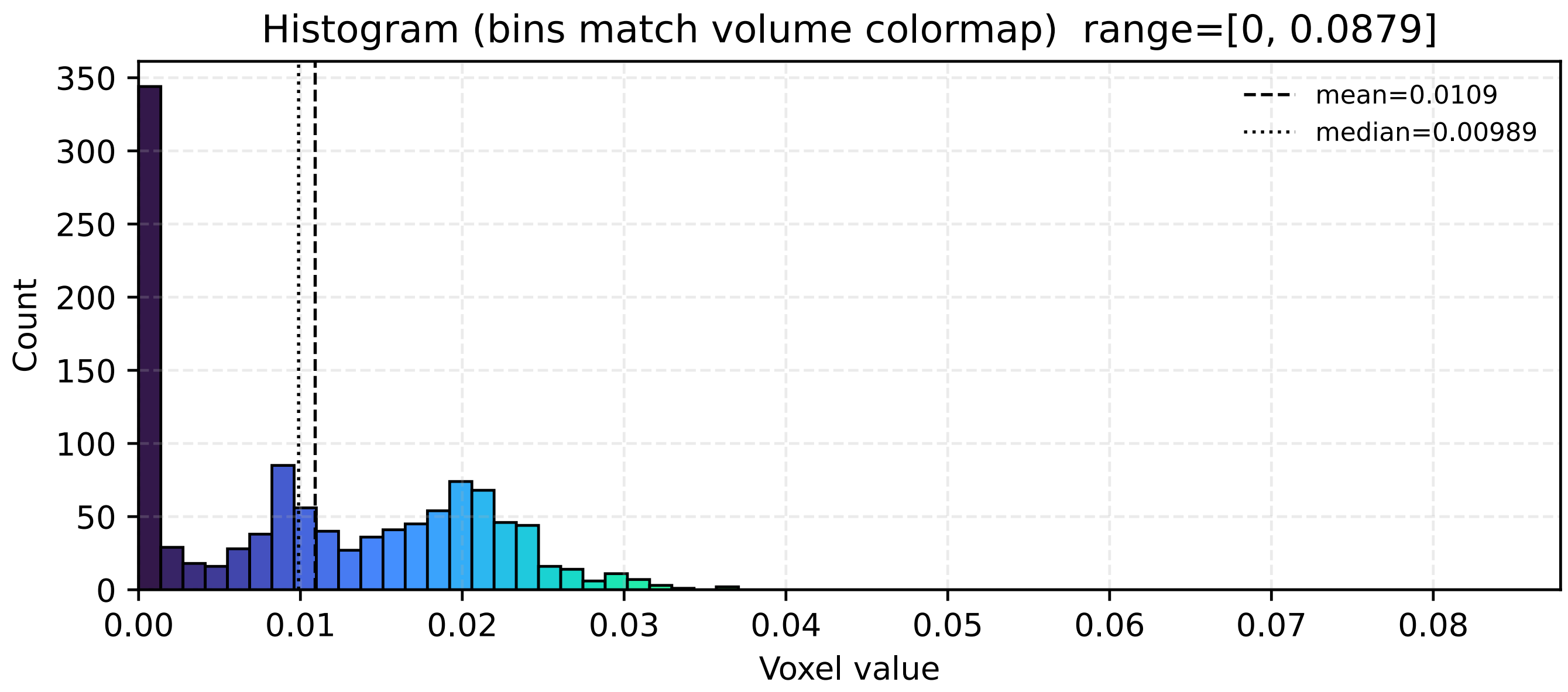

Lesser-improvement Participant in Testing Set

Axial slices from Participant 303673 shape=(79, 95, 79) [ROI: Temporal Fusiform Cortex, posterior division] [smart slices] [crop x:24-73, y:43-65] [manual cbar 0–0.0878677]

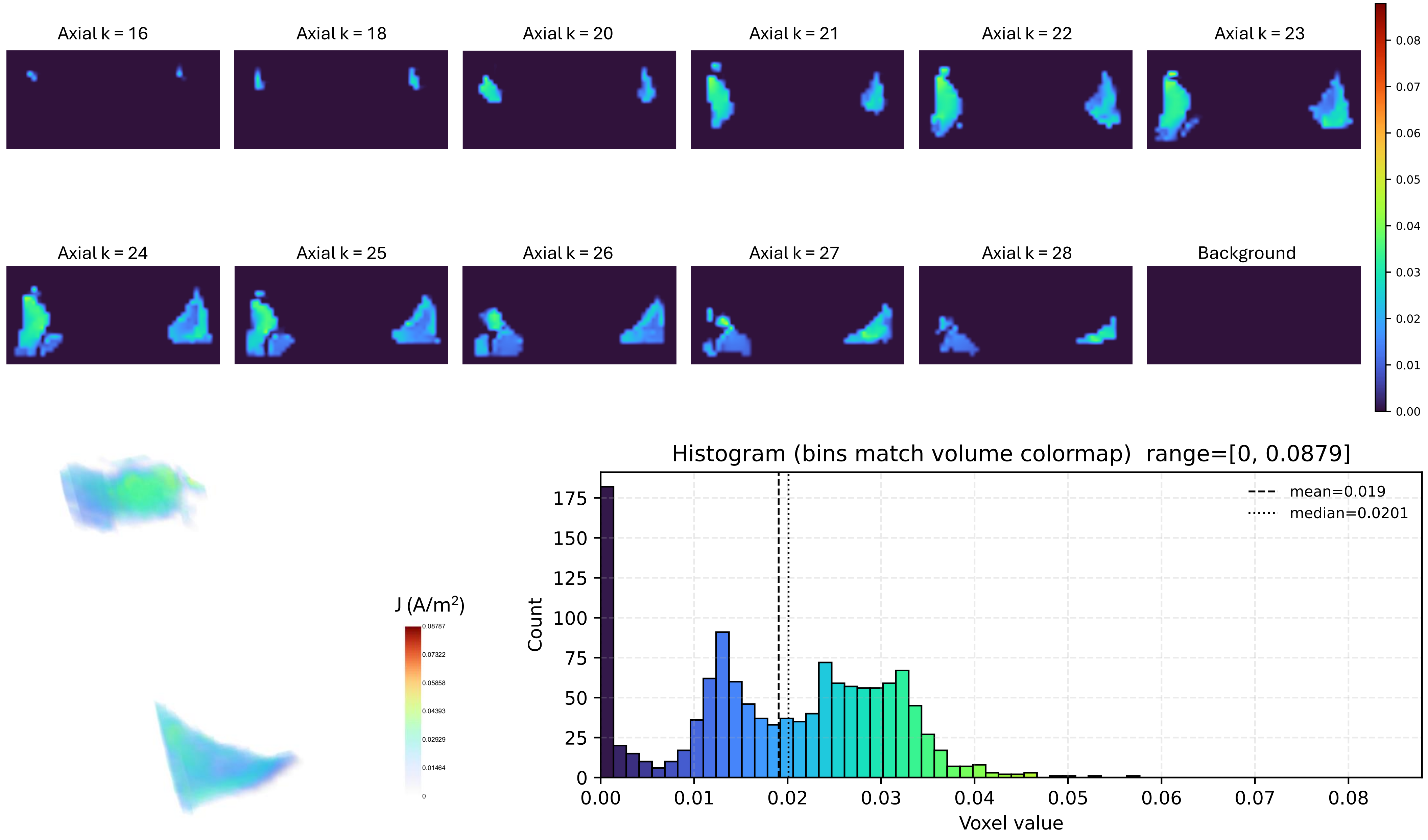

Supplement: Supplementary file 1 [file Data_Sheet_1.pdf]
