# Supplementary material for: Machine learning and individual variability in electrical field characteristics predict tDCS treatment response for anxiety in older adults in the ACT trial
Source: Front Hum Neurosci. 2026 Jul 31;20:1847131. doi: 10.3389/fnhum.2026.1847131 (PMC13473359; doi:10.3389/fnhum.2026.1847131)
Supplement: Supplementary file 2 [file Table_1.docx]

Supplementary Materials for “Machine Learning and Individual Variability in Electrical Field Characteristics Predict tDCS Treatment Response for Anxiety in Older Adults in the ACT Trial”

# 1 Data Characterization

## 1.1 Behavioral Response Characterization and Cohort Statistics

In a cohort of 20 participants with moderate/severe baseline state anxiety, greater-improvement participants ($n=11$) showed a large and consistent reduction in STAI state scores following intervention ($mean reduction = 21.9, SD = 6.2$), whereas lesser-improvement participants ($n=9$) exhibited minimal change ($mean reduction = 2.3, SD = 5.1$). This difference was highly significant ($Welch’s t = 6.93, p < 0.001$) with a very large effect size ($Hedges’ g > 3$). Similar patterns were observed within the University of the Florida cohort ($n=11$) and the University of Arizona cohort ($n=9$) sets: in both, greater-improvement participants demonstrated marked score reductions while lesser-improvement participants showed negligible score reductions. Effect sizes remained very large across training and testing sets, underscoring the reproducibility of the response classification. Baseline scores were comparable between groups, indicating that post-intervention divergence was not driven by initial severity but by differential responses. Descriptive and inferential statistics are presented in Table S1. At the University of Florida, participants 105256, 105971, 202808, 110081, 115991, and 202251 accepted 19, 18, 19, 13, 18, and 19 transcranial direct current stimulation sessions, respectively, while all other participants at the University of Florida and the University of Arizona accepted 20 transcranial direct current stimulation sessions.

Table S1: Descriptive and Inferential Statistics of STAI State Anxiety Scores for Full Cohort, Training, and Testing Sets. Welch’s t-test compared the mean changes in STAI state scores between greater-improvement participants and lesser-improvement participants in each cohort. State-Trait Anxiety Inventory (STAI); Standard Deviation (SD); Hedges’ effect size measure (Hedges’ g).

| Cohort | Group | Baseline STAI State Score Mean (SD) | Post STAI State Score Mean (SD) | Mean STAI State Score Reduction (SD) | Statistical Test | p-value | Effect Size (Hedges’ g) |
| --- | --- | --- | --- | --- | --- | --- | --- |
| Full Cohort  (n = 20) | Lesser-improvement ($n=9$) | 43.3 (4.3) | 41.0 (6.7) | 2.3 (5.1) | Welch’s t = 6.93 | <0.001 | 3.16 |
|  | Greater-improvement ($n=11$) | 45.7 (4.5) | 23.8 (3.6) | 21.9 (6.2) |  |  |  |
| Training Set  (n = 11) | Lesser-improvement ($n=5$) | 41.2 (2.9) | 37.8 (5.6) | –3.4 (5.6) | Welch’s t = 4.83 | 0.002 | 2.88 |
|  | Greater-improvement ($n=4$) | 43.8 (4.6) | 22.5 (3.1) | –21.3 (5.9) |  |  |  |
| Testing Set (n = 9) | Lesser-improvement ($n=4$) | 46.0 (4.5) | 45.0 (6.2) | –1.0 (4.9) | Welch’s t = 6.82 | <0.001 | 3.91 |
|  | Greater-improvement ($n=7$) | 46.9 (4.3) | 24.6 (3.9) | –22.3 (6.7) |  |  |  |

Regarding potential confounding variables, we performed greater-improvement versus lesser-improvement participant comparisons for demographic and clinical covariates, including baseline age, sex, marital status, race, ethnicity, height, weight, BMI, and years of education, stratified across the full cohort, training set, and testing set. For continuous variables, normality was assessed using the Shapiro–Wilk test, followed by Welch’s t-test or Mann–Whitney U test as appropriate; categorical variables were compared using Fisher’s exact test or chi-square tests. Across the full cohort, none of the evaluated covariates differed significantly between greater-improvement participants and lesser-improvement participants, including age, sex, BMI, and education. Similar nonsignificant patterns were observed within the training and testing sets, indicating that greater-improvement classification was not meaningfully confounded by measured demographic characteristics. These results are summarized in Table S2.

Table S2. Greater-improvement versus lesser-improvement comparison of demographic covariates in the full cohort, training set, and testing set.

| Cohort | Variable | Lesser-improvement | Greater-improvement | Test | p-value | Effect size |
| --- | --- | --- | --- | --- | --- | --- |
| Full Cohort | Age | 71.44 (5.53) | 75.09 (6.55) | Welch’s t-test | 0.1936 | 0.5711 |
| Full Cohort | Height | 67.11 (3.72) | 66.00 (3.41) | Welch’s t-test | 0.4999 | -0.2997 |
| Full Cohort | Weight | 162.33 (32.25) | 167.09 (20.26) | Welch’s t-test | 0.7067 | 0.1734 |
| Full Cohort | BMI | 25.15 (3.26) | 27.01 (3.31) | Mann–Whitney U | 0.4033 | 0.2323 |
| Full Cohort | Years of education | 16.22 (2.28) | 15.91 (2.47) | Welch’s t-test | 0.7718 | -0.1257 |
| Full Cohort | Sex | Female: 5; Male: 4 | Female: 8; Male: 3 | Fisher’s exact test | 0.6424 | 0.1791 |
| Full Cohort | Marital status | Married: 4; Divorced: 3; Widowed: 1; Single: 1 | Widowed: 5; Married: 4; Single: 1; Divorced: 1 | Chi-square test | 0.3205 | 0.4184 |
| Full Cohort | Race | White: 7; More than one race: 1; American Indian/Alaska Native: 1 | White: 10; Black or African American: 1 | Chi-square test | 0.3390 | 0.4101 |
| Full Cohort | Ethnicity | Not Hispanic or Latino: 8; Hispanic or Latino: 1 | Not Hispanic or Latino: 11 | Fisher’s exact test | 0.4500 | 0.2536 |
| Training Set | Age | 72.00 (7.16) | 76.43 (7.37) | Welch’s t-test | 0.3638 | 0.5546 |
| Training Set | Height | 66.75 (4.43) | 65.43 (3.51) | Welch’s t-test | 0.6294 | -0.3149 |
| Training Set | Weight | 167.50 (33.04) | 170.71 (17.98) | Welch’s t-test | 0.8659 | 0.1221 |
| Training Set | BMI | 26.31 (3.52) | 28.14 (3.53) | Mann–Whitney U | 0.7879 | 0.1429 |
| Training Set | Years of education | 15.00 (1.83) | 14.71 (1.70) | Mann–Whitney U | 0.9207 | -0.0714 |
| Training Set | Sex | Male: 2; Female: 2 | Female: 5; Male: 2 | Fisher’s exact test | 0.5758 | 0.2143 |
| Training Set | Marital status | Married: 2; Widowed: 1; Divorced: 1 | Married: 3; Widowed: 3; Single: 1 | Chi-square test | 0.4622 | 0.4837 |
| Training Set | Race | White: 3; More than one race: 1 | White: 7 | Fisher’s exact test | 0.3636 | 0.4183 |
| Training Set | Ethnicity | Not Hispanic or Latino: 3; Hispanic or Latino: 1 | Not Hispanic or Latino: 7 | Fisher’s exact test | 0.3636 | 0.4183 |
| Testing Set | Age | 71.00 (4.69) | 72.75 (4.79) | Welch’s t-test | 0.6008 | 0.3287 |
| Testing Set | Height | 67.40 (3.58) | 67.00 (3.46) | Welch’s t-test | 0.8703 | -0.1007 |
| Testing Set | Weight | 158.20 (34.84) | 160.75 (25.25) | Welch’s t-test | 0.9024 | 0.0729 |
| Testing Set | BMI | 24.21 (3.09) | 25.04 (1.83) | Welch’s t-test | 0.6340 | 0.2801 |
| Testing Set | Years of education | 17.20 (2.28) | 18.00 (2.31) | Mann–Whitney U | 0.7024 | 0.2000 |
| Testing Set | Sex | Female: 3; Male: 2 | Female: 3; Male: 1 | Fisher’s exact test | 1.0000 | 0.1581 |
| Testing Set | Marital status | Married: 2; Divorced: 2; Single: 1 | Widowed: 2; Married: 1; Divorced: 1 | Chi-square test | 0.3080 | 0.6325 |
| Testing Set | Race | White: 4; American Indian/Alaska Native: 1 | White: 3; Black or African American: 1 | Chi-square test | 0.3575 | 0.4781 |
| Testing Set | Ethnicity | Not Hispanic or Latino: 5 | Not Hispanic or Latino: 4 | NA | NA | NA |

## 1.2 Current Density Distribution (J-map) Characterization

Finite-element modeling was used to estimate the intracranial current density distribution (J-map) induced by transcranial direct current stimulation across brain tissues, including cerebrospinal fluid (CSF), gray matter (GM), and white matter (WM). Current density values were expressed in SI units ($A/m^{2}$). Across all valid brain voxels, the magnitude of current density ($J$) ranged from $1.78 \times10⁻⁴$ to $4.67 \times10⁻¹$ $A/m^{2}$, indicating substantial spatial heterogeneity in stimulation strength.

The NIfTI headers used an RAS+ coordinate convention with an identity affine (no axis flips/rotations), such that the x-, y-, and z-components correspond to the right–left, anterior–posterior, and superior–inferior directions, respectively (positive toward Right, Anterior, and Superior). Accordingly, $J_{x}$, $J_{y}$, and $J_{z}$ represent the projections of the current density vector onto these anatomical axes. Directional angles were derived from the electric field vector $\mathbf{E}=(E_{x},E_{y},E_{z})$using spherical coordinates. The inclination $\theta=arccos(E_{z}/\mid\mathbf{E}\mid)$quantified the angle relative to the positive superior axis (range $0$ to $\pi$), and the azimuth $\phi=arctan(E_{y},E_{x})$quantified the angle of the projection onto the axial (x–y) plane measured from +x toward +y (range $-\pi$ to $\pi$).

The component-wise distributions revealed predominantly negative values along the x-direction ($J_{x}$ range: $-0.359$ to $-1.16 \times10⁻⁴ A/m^{2}$), with more symmetric bidirectional distributions along the y- and z-directions ($J_{y}$ range: $-0.114$ to $0.103 A/m^{2}$; $J_{z}$ range: $-0.278$ to $0.135 A/m^{2}$). These results reflect anatomically constrained current flow shaped by tissue conductivity differences and head geometry, providing a quantitative basis for subsequent analyses linking individualized electric field exposure to behavioral and clinical outcomes. Across valid brain voxels, the electric field orientation spanned a broad angular distribution, with the inclination angle $\theta$ ranging from 0.44 to 2.88 radians (indicating directions from predominantly superior- to inferior-oriented fields) and the azimuth angle $\varphi$ covering the full range from $-\pi$ to $\pi$ radians, reflecting near-complete directional coverage within the axial plane.

# 2 Supplementary Method Details

## 2.1 Supervised Machine Learning Models

To predict whether an older adult with moderate/severe baseline state anxiety symptoms would be greater-improvement or lesser-improvement participant to the combined tDCS and CT intervention, we implemented a supervised machine learning framework tailored to the characteristics of neuroimaging-derived current density map data. The task was formulated as a binary classification problem, where the input consisted of participant-specific J-map and the output indicated greater-improvement versus lesser-improvement status.

The input to the supervised machine learning pipeline was a J-map derived from T1-weighted MRI scans. Prior to entry into the pipeline, the J-maps underwent a spatial normalization process to ensure anatomical comparability across participants. Specifically, masked J-map values were transformed to the Montreal Neurological Institute 152 template (MNI152) standard space using the International Consortium for Brain Mapping (ICBM) space template (European brains option) provided in the Statistical Parametric Mapping (SPM12 v6906, http://www.fil.ion.ucl.ac.uk/spm/) software package. SPM’s normalise function performed this transformation and applies both affine and nonlinear warping to align individual participant anatomy. The normalized J-maps thus provided a standardized spatial framework for feature extraction and subsequent analysis, reducing variability introduced by inter-subject differences in brain size and morphology.

To mitigate potential bias toward the majority (lesser-improvement) class, we employed the Synthetic Minority Oversampling Technique (SMOTE) only on training set, which generates synthetic minority samples in regions of the feature space where interpolation is deemed reliable. We implemented this approach using the open-source Python package called imbalanced-learn library (version 0.13.0; https://imbalanced-learn.org/). In our configuration, we used SMOTE with a sampling strategy of 1.0, ensuring equal representation between greater-improvement and lesser-improvement status. To maintain the local structure and reduce the likelihood of introducing noisy synthetic samples, we set the number of nearest neighbors of SMOTE for interpolation to 2, which constrains the generation of synthetic data to closely related points in the feature space.

To address the high dimensionality inherent in neuroimaging-derived features, we implemented a multi-stage feature reduction strategy that combined linear, statistical, and information-theoretic methods. First, we applied Principal Component Analysis (PCA), implemented in the scikit-learn Python library (version 1.6.1; https://scikit-learn.org). PCA reduced redundancy among features while retaining 95% of the explained variance, thereby preserving the most informative variance components. Next, we applied a Welch’s t-test filter, implemented through the SciPy package (version 1.16.0; https://scipy.org), to identify features that exhibited statistically significant group differences. We used a stringent significance threshold ($p < 1 \times10⁻⁴$) in the Welch’s t-test filter. Finally, we employed the Minimum Redundancy Maximum Relevance (mRMR) algorithm, implemented via the mrmr-selection Python library (version 0.2.8; https://github.com/smazzanti/mrmr). This step produced a compact subset of features that maximized relevance to the greater-improvement outcome while minimizing redundancy among themselves. Feature relevance was quantified by ranking each feature’s univariate association with the target using the F-test (ANOVA F-statistic), and redundancy was controlled through correlation-based penalization. As a result, 10–20 highly informative features were retained for subsequent modeling.

After feature selection, we introduced random Fourier feature mapping implemented in the scikit-learn. This step projected the selected features into a higher-dimensional space that approximates the radial basis function (RBF) kernel, enabling the model to capture nonlinear decision boundaries without the computational burden of explicit kernel methods. The final classification stage employed the logistic regression as classifier, also implemented in scikit-learn. This classifier performed linear classification in the nonlinear feature space generated by the Fourier feature mapping. Stochastic gradient descent was chosen with a maximum 1000 step size for the classifier training. The use of logistic regression classifier provided probabilistic outputs and yielded estimated likelihoods of tDCS + CT treatment response.

## 2.2 Machine Learning Model Training and Testing

We evaluated model generalizability using two distinct machine learning validation strategies: (1) a repeated mixed-site nested cross-validation approach and (2) a repeated cross-site validation, implemented separately to ensure the robustness and independence of performance estimates.

In the mixed-site approach, we conducted 20 independent repetitions of five-fold nested stratified cross-validation among participants who underwent the combined tDCS + CT treatment and exhibited moderate to severe baseline state anxiety, recruited from both the University of Florida and the University of Arizona. This mixed-site approach enabled estimation of model performance for data collected from different sites as a meta-analysis.

In the cross-site approach, participants who underwent the tDCS + CT intervention at the University of Florida were assigned to the training set, while those who underwent the intervention at the University of Arizona were assigned to the testing set. This site-based separation ensured that the testing set provided an independent evaluation of generalizability across distinct participant groups from a different site.

All model training was conducted on the training set, with hyperparameter optimization performed using Bayesian optimization (40 optimization iterations) coupled with three-fold cross-validation using only samples from the training set. Model selection was based on balanced accuracy. The Bayesian optimization was performed using a Gaussian Process (GP) surrogate model with a Matérn kernel (from the scikit-optimize package). The hyperparameters considered included the number of top features retained by the mRMR algorithm ($mRMR top-k = 10-500$), the width of the RBF kernel ($\gamma= {10}^{-3}-{10}^{1}$, log-uniform), and the number of random Fourier features used to approximate the RBF kernel ($n_{comp}= 100-1000$), while all other pipeline components were kept at their default settings. This strategy aimed to improve the ML model’s capacity to generalize while mitigating overfitting risks in the context of limited sample size.

To assess robustness and account for variability introduced by random initialization, the entire training and testing procedure was repeated independently under 40 distinct random seeds, resulting in 40 trained models. For each independent repetition, model predictions on the testing set were evaluated using a comprehensive suite of metrics, including balanced accuracy, precision, recall, weighted F1 Score, Matthews correlation coefficient (MCC), and receiver operating characteristic (ROC) and precision–recall (PR) curves. This repeated evaluation framework provided distributions of performance outcomes rather than single estimates, offering a more reliable assessment of model stability and predictive utility across different random conditions.

## 2.3 Voxel-wise Model Interpretation

To determine which brain regions most strongly influence our model’s predictions, we developed a post-hoc interpretation framework that generates voxel-level SHAP attributions in MNI152 space using the Python SHAP package (v0.42.1; <https://shap.readthedocs.io>). This approach preserves all transformations within the predictive pipeline while avoiding the computationally prohibitive task of calculating Shapley values at the raw voxel level. Direct computation of Shapley values for each voxel is infeasible, as it would require evaluating the model for all ($2^{V}+1$) possible subsets of ($V$) voxels. To address this, we restricted the analysis to the final stage of the pipeline. Specially, we calculated the SHAP values on the principal components selected by the Weltch’s t-test filters and the mRMR algorithm. These principal components were the inputs to the integrated models comprising the RBF mapping and the logistic regression classifier in our pipeline. After computing SHAP values for these principal components, we projected the SHAP values back onto the original voxels space in J-maps in MNI152 space, enabling voxel-wise interpretation of the model’s predictive contributions.

In order to focus on the spatial density distribution of absolute SHAP values across the brain identifying where SHAP importance is concentrated and use anatomical brain regions of interest (ROIs) to label and localize these high-density regions. We calculated the mean of the absolute SHAP values across voxels grouped by anatomical regions of interest (ROIs) defined by Harvard-Oxford atlas from Python Nilearn package (v0.12.1; <https://nilearn.github.io>), thereby resulting ROI-Level Spatial Density of SHAP Importance for each ROI. Specifically, we used the maximum probability atlases thresholded at 25% and resampled at 2 mm isotropic resolution (“cort-maxprob-thr25-2mm” and “sub-maxprob-thr25-2mm”), encompassing 48 cortical and 21 subcortical regions. Rather than summing voxel SHAP values within each ROI, ROI-Level Spatial Density of SHAP Importance normalized the sum of absolute SHAP values by voxel count to address differences in ROI size, as larger regions contain more voxels and thus more model inputs. Summing would bias the comparison by inflating the apparent importance of larger regions. By applying this normalization, ROI-Level Spatial Density of SHAP Importance reflects the average per-voxel contribution, independent of ROI volumes.

We instantiated a model-agnostic SHAP explainer with an independent masker, and a background distribution was established by randomly sampling 50 reference points (with replacement) from the test set ($n=9$), defining the baseline for feature marginalization. To examine the model’s decision behavior independently of specific subjects, we generated 100 synthetic test samples by introducing Gaussian noise to the feature space. SHAP values were then computed for these synthetic samples, quantifying how perturbations in individual features influenced the predicted probability of being a greater-improvement participant. This procedure produces spatially localized feature attributions that can be mapped back to brain space, facilitating neuroanatomical interpretation.

# 3 Supplementary Machine Learning Results Details

## 3.1 ROI-level spatial density of SHAP importance

Table S3 reports the ROI-level spatial density of absolute SHAP importance derived from our post-hoc interpretability pipeline. Model explanations were generated at the voxel level in MNI152 space using the Python SHAP package (version matching our environment) and subsequently aggregated within anatomical regions of interest (ROIs). ROI definitions were obtained from the Harvard–Oxford cortical and subcortical atlases, accessed through the Nilearn Python library (v0.12.1). Specifically, we used the deterministic maximum-probability atlas variants “cort-maxprob-thr25-2mm” for cortical parcellations and “sub-maxprob-thr25-2mm” for subcortical structures, both distributed via FSL and automatically downloaded by Nilearn (including totally 68 regions). These parcellations were used to compute the spatial density distribution of absolute SHAP values, quantifying the contribution of each anatomical region to the model’s predictions.

Table S3: ROI-level spatial density of absolute SHAP importance with non-zero values. Theses spatial density distribution of absolute SHAP values quantify how strongly each region contributes to the model’s predictions. Values are shown in units of$\times10⁻¹⁷$for readability. All remaining 34 ROIs not listed here exhibited ROI-level spatial density of absolute SHAP importance of zero, indicating no measurable contribution to the model’s prediction.

| ROI Name | ROI-level spatial density of absolute SHAP importance (× 10⁻¹⁷) |
| --- | --- |
| Cortex: Temporal Fusiform Cortex, posterior division | 2.658 |
| Cortex: Inferior Temporal Gyrus, posterior division | 2.443 |
| Cortex: Inferior Temporal Gyrus, temporooccipital part | 2.415 |
| Cortex: Supramarginal Gyrus, posterior division | 2.337 |
| Cortex: Lateral Occipital Cortex, inferior division | 2.263 |
| Cortex: Temporal Fusiform Cortex, anterior division | 2.157 |
| Cortex: Temporal Pole | 2.137 |
| Subcortex: Left Thalamus | 2.112 |
| Subcortex: Brain-Stem | 2.104 |
| Subcortex: Left Hippocampus | 2.101 |
| Cortex: Frontal Medial Cortex | 2.070 |
| Cortex: Lateral Occipital Cortex, superior division | 2.048 |
| Subcortex: Left Cerebral Cortex | 2.034 |
| Subcortex: Left Cerebral White Matter | 2.015 |
| Cortex: Insular Cortex | 2.013 |
| Cortex: Central Opercular Cortex | 2.005 |
| Subcortex: Right Cerebral Cortex | 2.003 |
| Cortex: Parahippocampal Gyrus, posterior division | 1.999 |
| Subcortex: Right Cerebral White Matter | 1.992 |
| Cortex: Paracingulate Gyrus | 1.964 |
| Cortex: Parahippocampal Gyrus, anterior division | 1.950 |
| Cortex: Middle Temporal Gyrus, anterior division | 1.939 |
| Cortex: Parietal Opercular Cortex | 1.918 |
| Cortex: Superior Parietal Lobule | 1.907 |
| Subcortex: Left Pallidum | 1.880 |
| Cortex: Postcentral Gyrus | 1.835 |
| Subcortex: Right Amygdala | 1.821 |
| Cortex: Frontal Orbital Cortex | 1.815 |
| Cortex: Angular Gyrus | 1.783 |
| Cortex: Occipital Fusiform Gyrus | 1.725 |
| Cortex: Precentral Gyrus | 1.710 |
| Subcortex: Left Lateral Ventricle | 1.676 |
| Cortex: Superior Frontal Gyrus | 1.578 |
| Subcortex: Left Putamen | 1.572 |

## 3.2 Current Density Maps in the Fusiform Cortex

We present voxels representing the magnitude of current density distribution in the temporal fusiform cortex, posterior division, shown in MNI space for each participant individually in a file titled Supplementary Figures. These maps illustrate individual differences in tDCS-induced current flow across the 20 participants.

## 3.3 Model Performance After Adding Confounder Variables

To assess whether J-map–based prediction was influenced by potential demographic and anthropometric confounders, we compared models using J-map features alone with models incorporating J-map features plus covariates including age, sex, marital status, race, height, weight, body mass index, and years of education. As shown in Table S4, adding confounder variables consistently reduced model performance across both mixed-site and cross-site validation. The J-map-only models outperformed the confounder-augmented models across all evaluation metrics, indicating that the predictive signal was not improved by inclusion of measured confounders. These findings suggest that J-map features retained stronger predictive value on their own and that the observed performance was not primarily driven by simple demographic or clinical covariates.

Table S4. Greater-improvement prediction performance using J-map features alone versus J-map features plus confounder variables

| Validation Strategy | Feature Input | AUPRC | AUROC | Balanced Accuracy | Weighted F1 | MCC |
| --- | --- | --- | --- | --- | --- | --- |
| Mixed-site stratified split | J-map only | 0.90 ± 0.10 | 0.89 ± 0.11 | 0.82 ± 0.16 | 0.81 ± 0.17 | 0.67 ± 0.29 |
|  | J-map + confounders | 0.48 ± 0.20 | 0.61 ± 0.15 | 0.49 ± 0.14 | 0.42 ± 0.16 | −0.02 ± 0.34 |
| Cross-site validation | J-map only | 0.73 ± 0.12 | 0.74 ± 0.12 | 0.72 ± 0.06 | 0.70 ± 0.06 | 0.47 ± 0.13 |
|  | J-map + confounders | 0.61 ± 0.16 | 0.66 ± 0.12 | 0.52 ± 0.09 | 0.36 ± 0.14 | 0.04 ± 0.25 |
